# Unlocking the Puzzle of Mammalian Transfection: The Role of the RNA-sensing-Mediated Interferon Response in the Cellular Defense Against Foreign DNA Intrusion

**DOI:** 10.7150/ijbs.107510

**Published:** 2025-06-09

**Authors:** Xiaoyu Li, Yicen Zhou, Jie Wei, Wei Sun, Ligang Fan, Jian Yan

**Affiliations:** 1 Ministry of Education Key Laboratory of Resource Biology and Biotechnology in Western China; Shaanxi Provincial Key Laboratory of Biotechnology; College of Life Sciences, Northwest University, Xi'an 710069, China.; 2 Department of Biomedical Sciences, Jockey Club College of Veterinary Medicine and Life Sciences; Tung Biomedical Sciences Centre, City University of Hong Kong, Kowloon Tong, Hong Kong SAR, China.; 3 Department of Precision Diagnostic and Therapeutic Technology, The City University of Hong Kong Shenzhen Futian Research Institute, Shenzhen 518016, China.; 4 Department of Urology, The First Affiliated Hospital of Xi'an Jiaotong University, Xi'an 710061, China.

**Keywords:** Transfection efficiency, innate immune response, RNA-sensing, epigenetic memory, IRF3/7, MDA5

## Abstract

The introduction of foreign DNA into mammalian cells to express a given gene or genes of interest is a pivotal process with significant implications for molecular biology and gene therapy. Despite the development of various methods to improve transfection efficiency, it remains suboptimal in many cell types. The cyclic GMP-AMP synthase (cGAS)-stimulator of interferon genes (STING) senses transfected DNA and elicits an innate immune response, leading to low transfection efficiency. However, the mechanism by which transgene expression is repressed by cGAS-STING activation remains elusive. In this study, we demonstrated the crucial involvement of multiple RNA processing pathways under the control of cGAS-STING-activated IRF3/7 in suppressing transgene expression. These pathways included RNA-sensing genes (e.g., MDA5 and RIGI), as well as the OAS family (mRNA degradation) and the IFIT family (translation inhibition). By depleting IRF3/7, cGAS-STING, or RNA-sensing genes, we observed a significant increase in the transfection efficiency of the treated cells, with the most pronounced effects observed in the STING and MDA5 double-knockdown group. Our findings provide insights into the interconnected roles of DNA- and RNA-sensing mechanisms in innate immune activation triggered by transgene expression, thereby suggesting potential strategies to increase transfection efficiency in biomedical research.

## Introduction

Transfection refers to the process of introducing foreign DNA into mammalian cells, enabling the expression of exogenous genes 1. This technique has broad applications in gene therapy, drug development, and molecular biology, including protein expression, gene editing, and the construction of cell models 2-9. Transfection efficiency is critical for the success of these applications. Various transfection methods have been developed, such as liposome-mediated transfection, electroporation, and chemical coprecipitation, which have improved the transfection efficiency to some extent 10. However, many cell types still exhibit significantly lower transfection efficiency than do HEK293T cells. Since a low transfection efficiency often poses a bottleneck in experimental workflows, enhancing both the efficiency of transfection and the expression of gene-carrying plasmids is of urgent importance in multiple research fields. Transfection typically involves several challenging steps, including cellular uptake, endosomal escape, and transport through the cytoplasm and into the nucleus 11. One key factor that substantially influences transgene expression is the stability of the transfected DNA or mRNA. When plasmid DNA is introduced into cells, cyclic GMP-AMP synthase (cGAS) is activated, catalyzing the production of cyclic GMP-AMP (cGAMP), which subsequently activates stimulator of interferon genes (STING) on the endoplasmic reticulum membrane. Activated STING then promotes the activation of TANK-binding kinase 1 (TBK1) and IκB kinase (IKK) 12. However, how activation of the cGAS-STING pathway ultimately leads to the suppression of transgene expression remains poorly understood.

In this study, we performed RNA sequencing to investigate the shared and distinct cellular responses to various circular and linear DNA molecules in five cell lines, namely, HEK293T, HCT116, HeLa, L02, and NCM460 cells, which exhibit striking differences in transfection efficiency. Our results indicated that the low transfection efficiency was attributable primarily to a strong innate immune response. We then explored key factors involved in regulating this response to plasmid DNA transfection. Notably, our findings revealed that mRNA processing could play a critical role in suppressing transgene expression. This regulatory network includes RNA-sensing genes such as MDA5 and RIGI; RNA degradation-associated genes such as OAS1/2/3/L; and translation-inhibiting genes such as IFIT1/2/3. Furthermore, we showed that inhibition of the RNA-sensing pathways and the cGAS-STING axis significantly increases transgene expression, highlighting the convergent roles of DNA- and RNA-sensing mechanisms in activating interferon-stimulated genes (ISGs) and repressing transgene expression. These insights may contribute to the development of broadly applicable strategies for improving transfection efficiency in biomedical research.

## Materials and Methods

### Cell culture

The HEK293T cell line was cultured in Dulbecco's modified Eagle's medium (DMEM; Gibco, 11995040) supplemented with 10% fetal bovine serum (FBS; Gibco, 10270106), 100 units/mL penicillin, and 100 µg/mL streptomycin (Gibco, 15140122). The HCT116 cells were maintained in McCoy's 5A modified medium (Gibco, 16600082) under identical conditions. HeLa cells were cultured in minimum essential medium (MEM; Gibco, 10370021) supplemented with 10% FBS, 100 units/mL penicillin, and 100 µg/mL streptomycin. L02 and NCM460 cells were grown in RPMI 1640 medium (Gibco, 11875093) supplemented with 10% FBS, 100 units/mL penicillin, and 100 µg/mL streptomycin. All the cell lines were incubated at 37 °C in a 5% CO_2_ atmosphere and authenticated to confirm that they were mycoplasma-negative.

### Cell transfection

The cells were transfected with jetPRIME transfection reagent (Polyplus, 101000046) or Lipomaster 3000 (Vazyme, TL301) according to the manufacturer's protocol. The plasmids used for transfection included pcDNA3.1-neo (our laboratory), pXR004:CasRX pre-gRNA cloning backbone (Addgene, 109054), pcDNA3.1(+) EGFP (Addgene, 129020), pGL4.23[luc2-minP] (Addgene, 226458), pGL4.74[hRluc/TK] (Promega, E6921), and pUC19 (Addgene, 50005). Additionally, the linear DNA constructs pcDNA3.1-neo (KpnI), CasRX pre-gRNA cloning backbone (EcoRI), and pcDNA3.1(+) EGFP (DraIII) were used. The transfection protocols were also applied to deliver siRNAs targeting specific genes, including sicGAS, siSTING, siMDA5, siRIGI, siIRF3, and siIRF7.

### Flow cytometry analysis

The cells transfected with the plasmid carrying the EGFP were washed with PBS and treated with 0.25% trypsin/EDTA. After gentle pipetting to obtain a single-cell suspension, the cells were centrifuged and resuspended in PBS at a concentration of 1×10^6^ cells/mL. The suspension was filtered through 50 µm nylon mesh before analysis. GFP-positive cells were quantified at 24 hours posttransfection using a FACS Aria III cell sorter (Becton, Dickinson, and Company). Data acquisition and analysis were conducted using FlowJo software (BD, California, USA).

### RT‒qPCR

Total RNA was extracted using the Universal RNA Extraction Kit (TaKaRa, 9767) according to the manufacturer's instructions and reverse transcribed into cDNA using the HiScript II Q RT SuperMix Synthesis Kit (Vazyme, R223). Quantitative real-time PCR (qPCR) was performed in triplicate using ChamQ Universal SYBR qPCR Master Mix (Vazyme, Q711) and a QuantStudio 3 Real-time PCR instrument (Thermo Fisher). Relative expression was normalized using the 2^-∆∆Ct^ method, and GAPDH was used as the reference gene. The relevant primers used are listed in **Table S3**.

### Western blot

The cells were lysed at room temperature for 30 minutes using 200 μL of RIPA buffer [140 mM NaCl, 1 mM EDTA (pH 8.0), 1% Triton X-100, 0.1% SDS, 0.1% sodium deoxycholate, and 10 mM Tris-HCl (pH 8.0)] containing protease inhibitors (Thermo Fisher, A32963) and phosphatase inhibitors (Beyotime, P1050). The protein concentration was measured using the Pierce™ BCA Protein Assay Kit (Thermo Fisher Scientific, 23227). Equal amounts of protein were resolved by SDS‒PAGE and immunoblotted with specific antibodies. For Western blot analyses, primary antibodies were diluted in 0.5% BSA in TBST, and secondary antibodies were diluted in 5% nonfat milk. Visualization and quantification of the Western blot signals were performed using the Bio-Rad ChemiDoc Imaging System and Fiji (v2.14.0). The antibodies used in this assay included anti-human cGAS (15102), anti-p-STING (Ser366, 50907), anti-IRF3 ( 4302), anti-p-IRF3 (Ser396, 4947), anti-TBK1 (3504), anti-p-TBK1 (Ser172, 5483), anti-MDA5 (5321), anti-RIGI (3743), and anti-IRF7 (4920) from Cell Signaling Technology; anti-STING (19851), GAPDH (10494), and EGFP (66002) from Proteintech; and anti-eIF2α (A21221), and anti-p-eIF2α (Ser51, AP0692) from ABclonal. HRP-labeled goat anti-mouse IgG (H+L) (Beyotime, A0216) and HRP-labeled goat anti-rabbit IgG (H+L) (Beyotime, A0208) served as secondary antibodies. Chemiluminescence was detected using the SuperSignal West Femto Maximum Sensitivity Substrate (Thermo Fisher Scientific, 34094).

### RNA-seq sequencing

Total RNA was extracted using the TAKARA MiniBEST Universal RNA Extraction Kit according to the manufacturer's guidelines. The RNA-Seq library was constructed using the ligation-mediated RNA sequencing (LM-seq) protocol 13 with three biological replicates. Initially, mRNA was isolated from the total RNA using the Next Poly A+ Isolation Kit (NEB), followed by reverse transcription with the Clontech SmartScribe Kit. The complementary DNA was purified using AMPure XP beads (Vazyme) before proceeding to adapter ligation and PCR amplification. Finally, the PCR products were purified and prepared for sequencing at BGI.

### ChIP-seq

ChIP-seq was performed according to established laboratory protocols 14. Briefly, the cells were crosslinked with 1% formaldehyde at room temperature for 10 minutes. The reaction was quenched with 125 mM glycine for 5 minutes. The cells were washed twice with cold PBS and treated with hypotonic lysis buffer (20 mM HEPES pH 7.9, 10 mM KCl, 1 mM EDTA, 10% glycerol, and 1 mM DTT, along with protease inhibitors (Roche)) to isolate the nuclei. Nuclei were resuspended in RIPA buffer (10 mM Tris-HCl pH 8.0, 140 mM NaCl, 1 mM EDTA, 1% Triton X-100, 0.1% SDS, 0.1% sodium deoxycholate, and protease inhibitors) and sonicated using Covaris M220 to achieve chromatin sizes of approximately 300-600 bp. The fragmented chromatin was precleared with Protein G agarose beads (GE Healthcare). The supernatant was diluted with RIPA buffer, and 30 μL was saved as the input. The remaining supernatant was incubated overnight at 4 °C with H3K27ac (Abmart, T59439), p-IRF3 (Abcam, 4947), and IRF7 (Abcam, 4920) antibodies. The next day, Protein G agarose beads preblocked with 0.5% BSA were added to the samples, which were subsequently incubated at 4 °C for 2 hours. The samples were washed sequentially with RIPA buffer, RIPA buffer containing 0.5 M NaCl, LiCl buffer [250 mM LiCl, 1 mM EDTA, 0.5% IGEPAL CA-630, 0.1% sodium deoxycholate, and 10 mM Tris-HCl (pH 8.0)], and Tris-EDTA (TE) buffer by gently rocking the beads for 5 min for each wash. The beads and input samples were added to 150 μL of extraction buffer [1% SDS in 1× TE, 12 μL of 5 M NaCl, and 10 μg of RNase A] and incubated at 37 °C for 1 hour. The cross-links in the protein‒DNA complexes were reversed by an overnight incubation at 65 °C with proteinase K. DNA was isolated through phenol/chloroform/isoamyl alcohol extraction and prepared for ChIP-seq library construction. Sequencing was conducted on the BGI platform.

### ChIP-seq data analysis

The ChIP-seq reads were aligned to the hg19 reference genome using Bowtie2 (v2.5.1) 15. Following alignment, duplicate reads were removed using the MarkDuplicates command from Picard (v3.1.1) 16. Peaks were then called using MACS2 (v2.2.9.1) 17. BAM files from biological replicates were merged using samtools (v1.17) 18 and converted to bigwig format with the bamCoverage command from deeptools (v3.5.1) 19, applying the -normalizeUsing RPKM option.

The normalization of the ChIP-seq data involved generating bigwig files of log_2_(IP/input) signal for cells treated with the transfection reagent alone, those transfected with the pcDNA3.1-neo plasmid, and those transfected with the linear pcDNA3.1-neo plasmid using bamCompare. Subsequently, bigwigCompare was used to generate log_2_(pcDNA3.1-neo/control) and log_2_(linear pcDNA3.1-neo/control) bigwig files. These files were further processed using the computeMatrix scale-regions command to generate matrices centered on differential peaks, with 8 kb extensions both upstream and downstream. Heatmaps were then created using plotHeatmap. The Integrative Genomics Viewer (IGV) was used to visualize ChIP signals, which were calculated using the bamCompare subtract option to normalize each sample to its input.

### Differential binding analysis and motif discovery

For the IRF7 and p-IRF3 ChIP-seq data from cells transfected with linear pcDNA3.1-neo or treated with the transfection reagent alone, differential peaks were identified using the bdgdiff command of MACS2 after the BAM files of three replicates were merged. An H3K27ac ChIP-seq peak differential binding analysis was performed using DiffBind (v3.8.4) 20, which compared the pcDNA3.1-neo- and linear pcDNA3.1-neo-transfected groups to the control group. Differentially upregulated peaks were selected based on a log fold change > 0 and FDR < 0.05.

Annotated peaks were obtained using ChIPseeker (v1.34.1) 21, focusing on differentially upregulated peaks in promoter regions (3 kb around the TSS). Motif discovery within these peaks was conducted using the findMotifs.pl program from HOMER 22, with promoter region peaks having a log fold change ≤ 0 serving as the background.

### RNA-seq and differential gene expression analyses

The raw RNA-Seq data were initially subjected to quality control using FastQC^23^ to ensure that high-quality reads were retained. These reads were then aligned to the human reference genome hg19, sourced from the GENCODE database, using HISAT2 (v2.1.0) 24. After alignment, featureCounts (v2.0.1) 25 was employed to quantify reads at the gene level, generating per-gene read counts for subsequent analysis.

DEGs were identified using DESeq2 (v1.42.1) 26, with the criteria set at a fold change ≥ 2 or ≤ -2 and an adjusted P value ≤ 0.05. Visualization of these DEGs was performed using the ggplot2 (v3.3.5) 27 and ComplexHeatmap (v2.10.0) 28 packages to generate volcano plots and heatmaps, respectively. Venn diagrams were generated using Evenn software. The Gene Ontology (GO) analysis was conducted using the clusterProfiler (v4.10.1) 29 package.

Normalized log_2_ fold change values of upregulated DEGs after transfection with circular or linear plasmids in all cell lines were subjected to PCA using the R function prcomp.

### Statistical analysis

**Figure 1:**
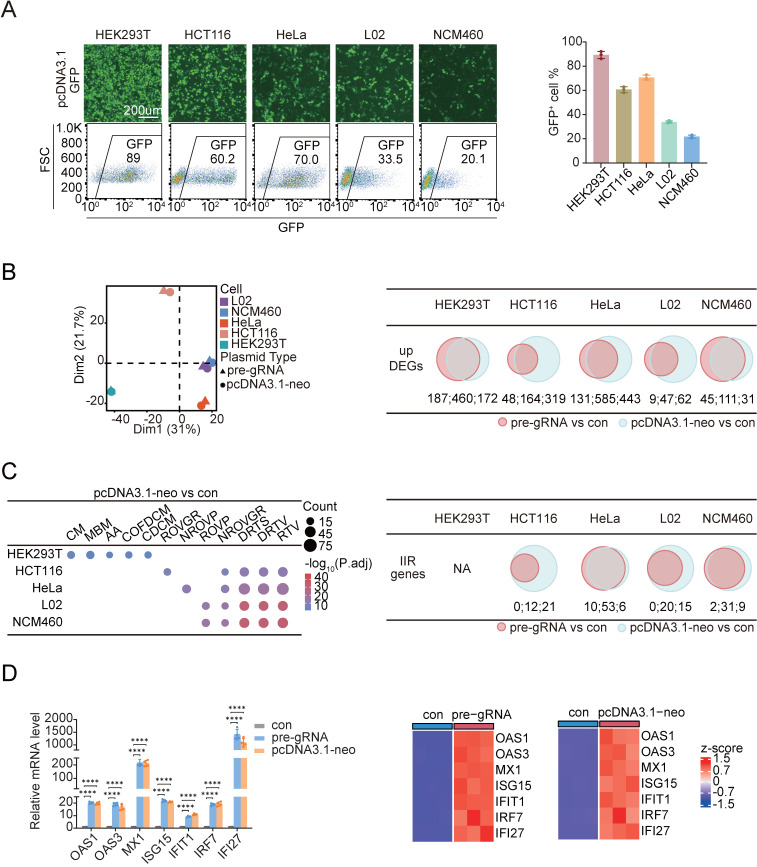
** The innate immune response reduces the transfection efficiency in mammalian cells.** (A) GFP fluorescence images and flow cytometry results (left panel) showing GFP-positive cell ratios (right panel) for HEK293T, HCT116, HeLa, L02, and NCM460 cells transfected with pcDNA3.1-GFP to assess transfection efficiency. FSC: forward scatter. n=3 independent replicates. (B) Principal component analysis of log_2_FC (left panel) and Venn diagrams (right panels) of upregulated DEGs identified in five cell lines following transfection with two circular plasmids. The upregulated DEGs identified in the pre-gRNA-transfected group are indicated by red circles, whereas the DEGs identified in the pcDNA3.1-neo-transfected group are indicated by blue circles. DEGs: differentially expressed genes. The numerical values corresponding to each Venn diagram region are annotated below and separated by colons. n=3 independent replicates. (C) Dot plots (left panel) display the top five most significantly enriched biological process GO terms among the upregulated DEGs in each cell line following pcDNA3.1-neo plasmid transfection compared with the controls. CM: cilium movement; MBM: microtubule-based movement; AA: axoneme assembly; COFDCM: cilium or flagellum-dependent cell motility; CDCM: cilium-dependent cell motility; ROVGR: regulation of viral genome replication; NROVP: negative regulation of viral process; ROVP: regulation of viral process; NROVGR: negative regulation of viral genome replication; DRTS: defense response to symbiont; DRTV: defense response to virus; RTV: response to virus. P.adj: adjusted p value. Venn diagrams (right panel) of IIR genes identified in the five cell lines following transfection with two circular plasmids are shown. Genes identified in the pre-gRNA-transfected group are indicated by red circles, whereas genes identified in the pcDNA3.1-neo-transfected group are indicated by blue circles. The numerical values corresponding to each Venn diagram region are annotated below and separated by colons. n=3 independent replicates. (D) qPCR analysis (left panel) of the expression of seven IIR genes in HeLa cells at 48 h after treatment with the transfection reagent or circular plasmids. Heatmaps (right panels) illustrate the differential expression levels of these genes between the control and circular plasmid-transfected groups. Con: control. Statistical P values were calculated using Dunnett's test after ANOVA, with con as the control group. *P < 0.05, **P <0.01, ***P < 0.001 and ****P < 0.0001. n=4 independent replicates.

**Figure 2:**
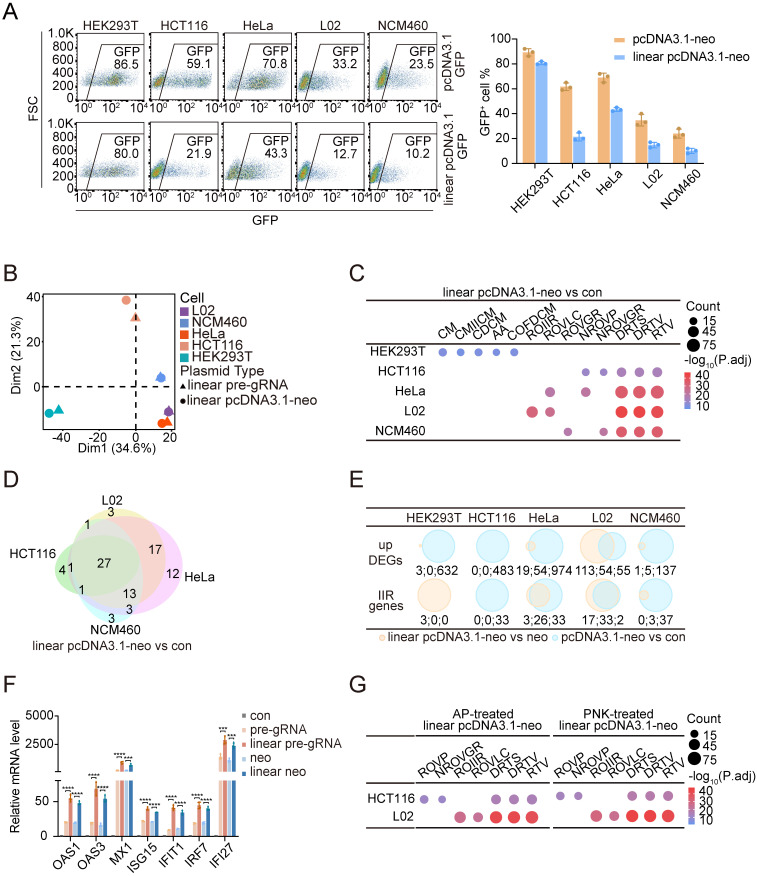
** Linear DNA triggers a stronger immune response than circular DNA.** (A) Flow cytometry results (left panel) and the ratios of GFP-positive cells (right panel) among HEK293T, HCT116, HeLa, L02, and NCM460 cells, which were used to assess the transfection efficiency of the pcDNA3.1-GFP and linear pcDNA3.1-GFP plasmids. FSC: forward scatter. n=3 independent replicates. (B) PCA of log2FC values between upregulated DEGs in the five cell lines following transfection with linear pre-gRNA or linear pcDNA3.1-neo plasmids. n=3 independent replicates. (C) Dot plots illustrating the top five representative GO biological process terms for upregulated DEGs identified in each cell line after transfection with the linear pcDNA3.1-neo plasmid compared with the control groups. CM: cilium movement; CMIICM: cilium movement involved in cell motility; CDCM: cilium-dependent cell motility; AA: axoneme assembly; COFDCM: cilium or flagellum-dependent cell motility; ROIIR: regulation of innate immune response; ROVLC: regulation of viral life cycle; ROVGR: regulation of viral genome replication; NROVP: negative regulation of viral process; NROVGR: negative regulation of viral genome replication; DRTS: defense response to symbiont; DRTV: defense response to virus; RTV: response to virus. P.adj: adjusted p value. n=3 independent replicates. (D) Venn diagram of IIR genes activated by the linear pcDNA3.1-neo plasmid compared with the transfection reagent alone across the HCT116, HeLa, L02, and NCM460 cell lines. Con: control. n=3 independent replicates. (E) Venn diagrams illustrating upregulated DEGs and IIR genes between linear pcDNA3.1-neo and pcDNA3.1-neo plasmid transfections (yellow) and pcDNA3.1-neo plasmid transfections and controls (blue). neo: pcDNA3.1-neo, con: control. The numerical values corresponding to each Venn diagram region are annotated below and separated by colons. n=3 independent replicates. (F) qPCR results at 48 h revealing the relative mRNA expression levels of 7 IIR genes in HeLa cells treated with either the transfection reagent alone or those transfected with circular or linear plasmids. neo: pcDNA3.1-neo. Statistical P values were calculated using two-tailed Student's t tests. For the groups transfected with linear pre-gRNA and linear pcDNA3.1-neo plasmids, the corresponding circular plasmid transfection group served as the control. *P < 0.05, **P <0.01, ***P < 0.001 and ****P < 0.0001. n=4 independent replicates. (G) Dot plots display the top five representative GO biological processes among the upregulated DEGs identified in HCT116 and L02 cells transfected with linear pcDNA3.1-neo plasmids treated with alkaline phosphatase (left panel) or polynucleotide kinase (right panel) compared with controls. ROVP: regulation of viral process; NROVGR: negative regulation of viral genome replication; ROIIR: regulation of innate immune response; ROVLC: regulation of viral life cycle; DRTS: defense response to symbiont; DRTV: defense response to virus; RTV: response to virus; AP: alkaline phosphatase. PNK: polynucleotide kinase. P.adj: adjusted p value. n=3 independent replicates.

All the statistical analyses were performed using GraphPad Prism software (version 10.1.2). For two-group comparisons, including the data in Figure 2F, Figure S4, Figure S5B, Figure S8A and B, Figure S11D and E, and Figure S12E, two-tailed Student's t test was used, whereas for multiple-group comparisons, including the data in Figure 1D, Figure 4B, D and E, Figure S1A and B, Figure S8D, Figure S10B, Figure S11A-C, and F, Figure S12A-D and F, Figure S13A, B, D and E, Figure S14B, Figure S15, Figure S16C-G, and Figure S17A and C, one-way analysis of variance (ANOVA) followed by Dunnett's test were used.

## Results

### Cellular defense mechanisms reduce transgene expression efficiency

We transfected the plasmid pcDNA3.1-GFP, which encodes enhanced green fluorescent protein (GFP) under the control of the constitutively active CMV promoter, using a commercially available liposome-based reagent to assess the transfection efficiency across different cell types. The transfection efficiency was evaluated by fluorescence microscopy and flow cytometry. We tested five commonly cultured human cell lines, namely, three normal epithelial cell lines, HEK293T (an embryonic kidney cell line), NCM460 (a normal adult colon mucosal epithelial cell line), and L02 (a normal hepatic cell line); and two tumor-derived cell lines, HCT116 (a colorectal adenocarcinoma cell line) and HeLa (a cervical adenocarcinoma cell line). Among these, HEK293T cells presented the strongest GFP fluorescence intensity per cell, indicating the highest level of transgene expression. The quantification of GFP-positive cells by flow cytometry further confirmed that HEK293T cells achieved the highest transfection efficiency, with approximately 90% of the population expressing detectable GFP (**Figure 1A**). In contrast, only ~20% of NCM460 cells expressed GFP, reflecting a much lower transfection efficiency. Interestingly, when plasmid uptake and expression were compared at various time points posttransfection, HEK293T cells presented one of the lowest levels of plasmid DNA uptake among all the tested cell lines (**Figure S1A**), despite their highest GFP expression (**Figure S1B**). Conversely, other cell types that took up greater amounts of plasmid DNA presented comparatively lower GFP expression (**Figure S1A**,** B**). This observation excluded the possibility that the different expression levels among the cell lines were caused by variations in the entry efficiency of foreign DNA into target cells.

We performed whole-transcriptome RNA sequencing (RNA-Seq) on cells transfected with different plasmid DNAs, including a pre-gRNA and a modified pcDNA3.1 plasmid lacking the neomycin resistance gene (pcDNA3.1-neo), to elucidate the mechanisms underlying the distinct transfection efficiencies observed among different cell lines (see **Figure S1C** for details). This approach enabled us to assess whether differences in transfection efficiency stemmed from the plasmid DNA sequences themselves or from the transgenes they encoded. We included a control group using the same transfection procedure without adding any DNA to the transfection mixture to avoid the influence of the transfection agents. As a result, we identified differentially expressed genes (DEGs) in each cell line following transfection with either pre-gRNA or pcDNA3.1-neo compared with the corresponding control group (**Figure S2**). In all cases, the number of upregulated genes substantially exceeded the number of downregulated genes. Using a consistent cutoff of |fold change| ≥ 2 and adjusted P value ≤ 0.05, we found that HEK293T cells presented the greatest number of DEGs, likely because of the high proportion of cells that successfully received the plasmid DNA and expressed the transgenes. Following cross-sample normalization, principal component analysis (PCA) was conducted to assess the similarity in cellular responses to transfection. The results indicated that HEK293T cells displayed a notably distinct phenotype compared to the other cell types (**Figure 1B**). We then sought to determine which biological pathways were most responsive to plasmid DNA transfection. The upregulated DEGs obtained for each cell type and different plasmid transfections were subjected to a Gene Ontology (GO) analysis. Strikingly, all cell types except HEK293T cells, regardless of their tissue of origin or stage of cancer derivation, exhibited the highly significant and reproducible enrichment of genes associated with “response to virus (RTV)”, “defense response to virus (DRTV)”, and related terms, even though the number of genes activated in different cells differed. In contrast, the top five pathways activated in HEK293T cells upon plasmid DNA transfection included “cilium movement”, “microtubule-based movement”, “axoneme assembly”, “cilium or flagellum-dependent cell motility”, and “cilium-dependent cell motility”, all of which are related to the increased motility of cells instead of innate immunity (**Figure 1C**). Notably, low STING expression in HEK293T cells may limit their ability to sense cytosolic DNA and mount an interferon-mediated antiviral response 30. The distinct transcriptional responses observed between HEK293T cells and the other cell types were consistent across both plasmids tested: pcDNA3.1-neo and pre-gRNA (**Figure 1C** and **Figure S3A**). Among the different cell lines, HeLa cells presented the activation of the greatest number of innate immune response (IIR) genes related to the GO term “RTV”, irrespective of the plasmid used (**Figure S3B**). We validated the activation of interferon-stimulated genes (ISGs)^31^ by selecting seven representative IIR-related genes, including OAS1, OAS3, MX1, ISG15, IFIT1, IRF7, and IFI27, for a quantitative real-time PCR (qPCR) analysis in HeLa cells, the results of which were consistent with those from RNA-Seq (**Figure 1D**). Previous studies have reported that the CMV promoter in vectors such as pcDNA3.0 may drive the low-level expression of small RNAs 32. We transfected cells with RNase A-treated pUC19, a bacterial vector lacking eukaryotic promoters, and monitored the expression of IIR-related genes to exclude the possibility that innate immune activation was due to RNA leakage from eukaryotic plasmids (**Figure S4**). As expected, pUC19 still elicited the significant upregulation of these genes. Taken together, these results suggested that the cellular defense mechanisms (i.e., IIRs) predominantly caused a difference in the efficiency of transgene expression in cultured human cells.

### Linear DNA is superior to circular DNA in inducing cellular immune responses

We next asked whether the cellular responses differed between cells transfected with circular or linearized plasmid DNA. We investigated these responses by linearizing the pcDNA3.1-neo and pre-gRNA plasmids using the type II restriction endonucleases KpnI and EcoRI, respectively. Interestingly, in HCT116, HeLa, L02, and NCM460 cells, transfection efficiency was significantly reduced when linearized plasmid DNA was used compared with their circular counterparts. In contrast, no significant difference in the transfection efficiency was observed between linear and circular DNA forms in HEK293T cells (**Figure 2A** and **Figure S5A**). Furthermore, we confirmed that the reduced efficiency observed in NCM460 cells was not due to differences in DNA uptake between the circular and linear plasmids (**Figure S5B**). These observations prompted us to examine whether linear DNA induced a stronger innate immune response than circular DNA. We addressed this question by conducting an RNA-Seq analysis of all five cell lines transfected with either linear pre-gRNA or linear pcDNA3.1-neo plasmids and collected samples at 48 hours posttransfection (**Figure S6**). PCA revealed a similar discrepancy in the cellular response of HEK293T cells compared with that of the other cell types when exposed to linear DNA, consistent with our previous observations after plasmid transfection (**Figure 2B**). The GO analysis of the DEGs further confirmed these findings. For all the cell lines except for HEK293T cells, the pathways most significantly enriched in response to the transfection of linear DNA were RTV and related terms. In contrast, DEGs in HEK293T cells remained enriched in motility-related pathways (**Figure 2C** and **Figure S5C**). We further examined the overlap of IIR genes induced by linear DNA transfection. Notably, 26 genes were consistently upregulated in all five cell lines following linear pre-gRNA transfection, and 27 genes were shared across all the cell lines transfected with linear pcDNA3.1-neo plasmids (**Figure 2D**, **Figure S5D; Table S1**).

We next directly compared the changes in gene expression elicited by the transfection of DNA in linear versus circular form within the same cell type. Although the number of DEGs was limited, IIR genes remained consistently upregulated in most cell lines. Interestingly, even HEK293T cells activated a small subset of IIR genes when they were transfected with linear plasmids (**Figure 2E** and **Figure S5E**), further supporting the heightened immunostimulatory potential of linearized DNA. We validated these transcriptomic findings by examining the expression of seven previously analyzed IIR genes, i.e., OAS1, OAS3, MX1, ISG15, IFIT1, IRF7, and IFI27, through qPCR validation. The results revealed that the expression of these genes was significantly increased in cells transfected with linear DNA compared with those transfected with circular plasmids of the same sequence (**Figure 2F**). One major structural difference between linear and circular DNA is the presence of exposed 5'-phosphate or 3'-hydroxyl groups on the backbone of linear DNA. As a method to assess whether these terminal groups contribute to enhanced immune activation, we treated linear DNAs with alkaline phosphatase (AP) to remove phosphate groups or with polynucleotide kinase (PNK) to phosphorylate the DNA ends. However, treatment with either enzyme did not significantly alter IIR gene expression in HCT116 or L02 cells (**Figure 2G** and **Figure S5F**), suggesting that 5'-phosphorylation was unlikely to be responsible for the increased immune activation observed upon linear DNA transfection. These results collectively indicated that circular and linear DNA molecules could activate the innate immune defense and suppress transgene expression, but linear DNA provoked a markedly stronger response. Further investigation is needed to identify the specific molecular features responsible for the differential immunogenicity between linear and circular DNA.

### Transgenesis stimulates IRF3/7 and RNA sensors

**Figure 3:**
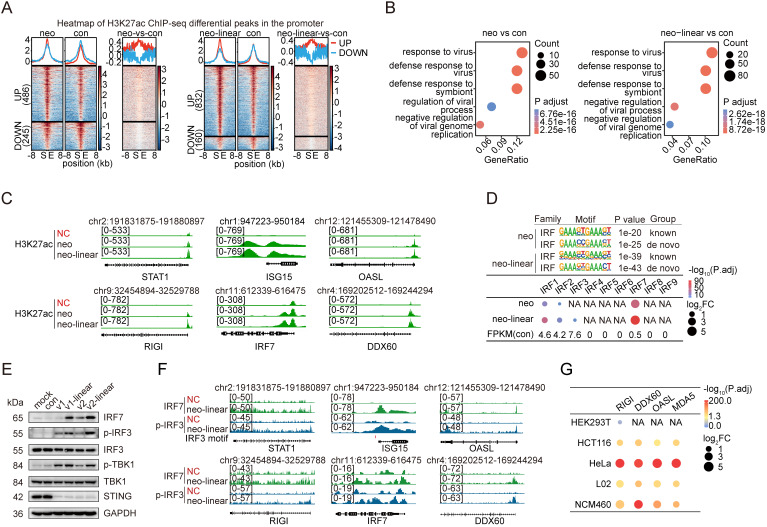
** Activation of IRF3/7 and RNA sensors in low-transfection-efficiency cells.** (A) Averaged profiles (top panels) and heatmaps (bottom panels) of H3K27ac ChIP-seq signals within the ±8 kb regions flanking the differential peaks on the promoters identified from HeLa cells transfected with circular or linear pcDNA3.1-neo plasmids compared with the control. The counts of upregulated and downregulated peaks are displayed on the left side of the heatmaps. Con: control. S: peak start site. E: peak end site. UP: upregulated peaks. DOWN: downregulated peaks. neo: pcDNA3.1-neo, neo-linear: linear pcDNA3.1-neo. n=3 independent replicates. (B) GO results for genes with active promoter regions containing upregulated differentially abundant ChIP-seq peaks in HeLa cells following transfection with pcDNA3.1-neo (left panel) or linear pcDNA3.1-neo (right panel) plasmids compared with the control groups. Con: control. P adjust: adjusted p value. neo: pcDNA3.1-neo, neo-linear: linear pcDNA3.1-neo. n=3 independent replicates. (C) H3K27ac ChIP-seq tracks of the STAT1, ISG15, OASL, RIGI, IRF7, and DDX60 genes in HeLa cells treated with only the transfection reagent or transfected with pcDNA3.1-neo or linear pcDNA3.1-neo plasmids. NC: negative control, neo: pcDNA3.1-neo, neo-linear: linear pcDNA3.1-neo. n=3 independent replicates. (D) The most significant Homer motifs (top panel) of the IRF transcription factor family from the known or de novo motif enrichment results, along with dot plots (bottom panels), illustrating the differential expression levels of all genes in the IRF family in HeLa cells transfected with pcDNA3.1-neo or linear pcDNA3.1-neo plasmids compared with the controls. Only data for genes with increased expression are shown. The average FPKM values of the IRF family genes in the control group are indicated at the bottom. P.adj: adjusted p value. FC: fold change. neo: pcDNA3.1-neo, neo-linear: linear pcDNA3.1-neo. n=3 independent replicates. (E) Western blot analysis of HeLa cells at 24 hours posttransfection with pre-gRNA, linear pre-gRNA, pcDNA3.1-neo, and linear pcDNA3.1-neo plasmids, showing the levels of IRF7, p-IRF3, IRF3, p-TBK1, TBK1, STING, and GAPDH. v1: pre-gRNA. v2: pcDNA3.1-neo. Con: control groups treated with only the transfection reagent. mock: wild-type cells. (F) IRF7 and p-IRF3 ChIP-seq tracks of the STAT1, ISG15, OASL, RIGI, IRF7, and DDX60 genes in HeLa cells treated with only the transfection reagent or transfected with linear pcDNA3.1-neo plasmids. The IRF3 motif region is marked in red. NC: negative control; neo-linear: linear pcDNA3.1-neo. n=3 independent replicates. (G) Dot plots illustrating the differential expression levels of RIGI, DDX60, OASL, and MDA5 across the five different cell lines following transfection with the pcDNA3.1-neo plasmid. n=3 independent replicates.

Epigenetic alterations enable cells to dynamically regulate gene expression in response to environmental stimuli 33, 34. We performed chromatin immunoprecipitation followed by deep sequencing (ChIP-seq) using a ChIP-grade antibody against acetylated histone H3 lysine 27 (H3K27ac), an epigenetic marker of active cis-regulatory elements, to investigate the mechanism underlying the activation of innate immune response (IIR) genes following the transfection of foreign DNA. The experiments were conducted in HeLa and NCM460 cells, each independently transfected with circular or linearized plasmid DNA. Cells subjected to the same transfection procedure without the addition of plasmid DNA were used as controls to identify significantly enriched loci marked by H3K27ac, referred to hereafter as “H3K27ac peaks”. In HeLa cells, more than 80,000 H3K27ac peaks were detected 24 hours after transfection with either the circular or linearized pcDNA3.1-neo plasmid. Similarly, approximately 70,000 peaks were obtained in NCM460 cells transfected with the same constructs. We identified significantly upregulated peaks (FDR < 0.05) using DiffBind^35^ to compare cells transfected with circular or linear pcDNA3.1-neo with control cells. Overall, 732 and 1,697 upregulated H3K27ac peaks were detected in HeLa cells that were unique to cells transfected with circular and linear pcDNA3.1-neo plasmids, respectively (**Table S2**), of which 486 (for circular DNA transfection) and 832 (for linear DNA transfection) peaks were located within the promoter region, defined as the 3 kb region flanking the transcription start sites (TSSs) of genes (**Figure 3A**). In addition, NCM460 cells presented significantly greater counts, with 1,969 and 1,166 upregulated H3K27ac peaks in cells transfected with circular and linear plasmid DNA, respectively (**Figure S7A**). A GO analysis was conducted for genes whose promoter regions contained one of these peaks. As expected, genes involved in the “response to virus” were essentially the most enriched in both cell types, regardless of circular or linear DNA transfection (**Figure 3B** and **Figure S7B**), as illustrated in the genome browser for representative IIR genes, i.e., STAT1, ISG15, OASL, RIGI, IRF7 and DDX60 (**Figure 3C** and **Figure S7C**; **Table S1**).

Epigenetic modifications are orchestrated by a central player known as the master transcription factor (TF)^36^. We performed a motif analysis of H3K27ac ChIP-seq peaks induced by transfected DNA to investigate the transcriptional regulators involved in the innate immune response to foreign DNA. Strikingly, transcription factors (TFs) belonging to the interferon regulatory factor (IRF) family were the most significantly enriched across both HeLa and NCM460 cells (**Figure 3D** and **Figure S7D**), suggesting that IRF family TFs are key mediators of the cellular defense response to transgene expression. Despite the high sequence similarity among the DNA-binding motifs of the nine IRF family members 37, 38, IRF7 exhibited markedly greater transcriptional upregulation than any other IRF TF in cells transfected with either circular or linear DNA and in both cell types tested (**Figure 3D** and **Figure S7D**).

cGAS-STING signaling pathway plays a central role in innate defenses, in which cGAS serves as a primary cytosolic DNA sensor that engages STING to initiate the interferon response 12, 39. Therefore, we examined the levels of proteins involved in the cGAS-STING pathway 40 with Western blotting, including IRF7, IRF3, p-IRF3 (phospho-IRF3 Ser386, 4D4G), p-TBK1 (phospho-TBK1/NAK Ser172, D52C2), TBK1, and STING, in cells transfected with circular or linear DNA for 24 hours. Notably, IRF7, p-IRF3, and p-TBK1 were simultaneously activated by the transfection of both types of DNA, particularly the linear DNA (**Figure 3E**), supporting our hypothesis that cGAS-STING signaling in the presence of foreign DNA in the cytosol would activate p-IRF3 and IRF7, consequently driving the transcription of interferon response genes. Moreover, we performed p-IRF3 and IRF7 ChIP-seq assays in HeLa cells transfected with the linear pcDNA3.1-neo plasmid, which revealed the binding of both TFs to the promoters of the aforementioned 6 representative IIR genes (**Figure 3F**).

We examined the genes whose promoters were bound by IRF7 and p-IRF3 and intersected them with IIR genes commonly activated in cell lines with low transfection efficiency (HCT116, HeLa, L02, and NCM460) upon plasmid DNA transfection to identify the downstream genes responsible for the cGAS-STING-mediated silencing of transgenes. Interestingly, multiple RNA-processing genes, including RIGI, DDX60, and OASL, emerged among the 6 genes identified at the intersection, suggesting that RNA sensing and processing might be involved in the cGAS-STING activation-induced silencing of transgenes. Although we did not detect the well-known RNA-sensing factor MDA5 in our list, the data clearly indicated its significant upregulation when cells were transfected with the pcDNA3.1-neo plasmid, especially in HeLa and NCM460 cells (**Figure 3G**). Moreover, silencing IRF3 or IRF7 in NCM460 cells reduced both the mRNA and protein levels of the two RNA sensors MDA5 and RIGI, further suggesting that they are potential targets of the cGAS-STING pathway (**Figure S8A-C**). IRF3 and IRF7 are known downstream factors of RNA sensors 41, 42, a finding that we confirmed previously (see **Figure S8D**). Collectively, our results suggest that IRF3 and IRF7 create a positive feedback loop that enhances RNA sensing while suppressing transgene expression.

Earlier studies have shown that these two RNA sensors can recognize viral double-stranded RNA, which in turn induces the interferon response 43, 44. The activation of these RNA sensors may be attributed to plasmid DNA undergoing rolling circle transcription, leading to the formation of complementary dsRNAs 43, 45. We performed Western blot analyses of key proteins in these sensing pathways at multiple time points in HeLa cells to clarify the temporal order of cGAS-STING and RNA sensors activation during the innate immune response to plasmid DNA. The results showed that the cGAS-STING-TBK1 axis was activated as early as 2 hours posttransfection, whereas RIGI/MDA5 activation did not occur until 8 hours posttransfection, suggesting that DNA sensing was activated prior to RNA sensing (**Figure S9**). This result was likely because transcription did not occur immediately upon DNA entry into the cells, and the initiation of RNA sensor transcription depended on IRF3, which was subsequently enhanced by the activation of the cGAS-STING-mediated DNA-sensing pathway. Regarding the dynamics of p-IRF3 and IRF7 activation, we observed that IRF3 phosphorylation was initiated at 2 hours posttransfection but was transient (**Figure S9**). In contrast, IRF7 expression began at approximately 16 hours posttransfection and peaked at 24 hours, indicating that IRF7 plays a role in the later stage of the cellular immune response. This delayed activation of IRF7 could be attributed to its role as a target gene of p-IRF3 (**Figure 3F**).

### Depleting DNA and RNA sensors improves the transfection efficiency

**Figure 4:**
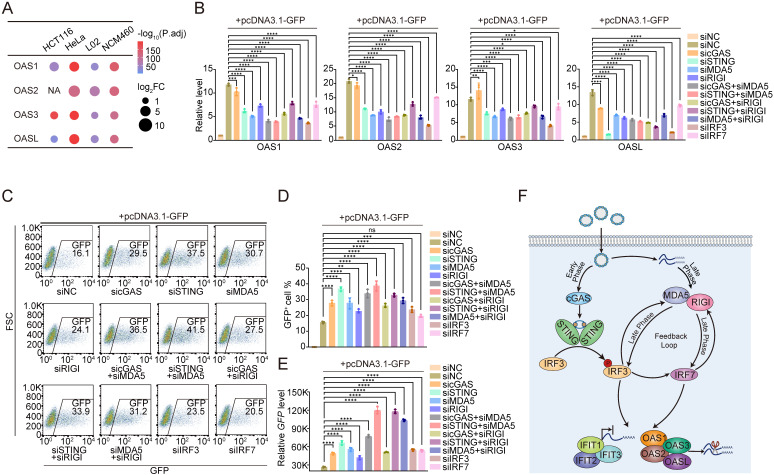
** Targeting IRF3/7 and DNA and RNA sensors to increase the transfection efficiency in host cells.** (A) Dot plots showing the differential expression levels of the OAS1, OAS2, OAS3, and OASL genes in HCT116, HeLa, L02, and NCM460 cells following transfection with the pcDNA3.1-neo plasmid. n=3 independent replicates. (B) qPCR results at 24 h revealing the relative OAS1, OAS2, OAS3, and OASL expression levels compared with those of the GAPDH gene after the knockdown of cGAS, STING, MDA5, RIGI, IRF3, and IRF7, as well as the double knockdown of factors in the DNA- and RNA-sensing pathways in NCM460 cells transfected with pcDNA3.1-GFP plasmids. Statistical P values were calculated using Dunnett's test after ANOVA, with siNC cells transfected with pcDNA3.1-GFP used as the control group. siNC: negative control siRNA. *P < 0.05, **P < 0.01, ***P < 0.001 and ****P < 0.0001. ns, not statistically significant. n=3 independent replicates. (C) Flow cytometry results of NCM460 cells transfected with pcDNA3.1-GFP plasmids following the knockdown of cGAS, STING, MDA5, RIGI, IRF3, and IRF7, as well as the double knockdown of factors in the DNA- and RNA-sensing pathways. NC: negative control. FSC: forward scatter. n=3 independent replicates. (D) GFP^+^ cell ratio determined by flow cytometry, as shown in Figure 4C. Statistical P values were calculated using Dunnett's test after ANOVA, with siNC cells transfected with pcDNA3.1-GFP used as the control group. siNC: negative control siRNA. *P < 0.05, **P < 0.01, ***P < 0.001 and ****P < 0.0001. ns, not statistically significant. n=3 independent replicates. (E) qPCR results at 24 h revealed the relative GFP expression levels compared with those of the GAPDH gene after the knockdown of cGAS, STING, MDA5, RIGI, IRF3, and IRF7, as well as the double knockdown of factors in the DNA- and RNA-sensing pathways in NCM460 cells transfected with pcDNA3.1-GFP plasmids. Statistical P values were calculated using Dunnett's test after ANOVA, with siNC cells transfected with pcDNA3.1-GFP used as the control group. siNC: negative control siRNA. *P < 0.05, **P < 0.01, ***P < 0.001 and ****P < 0.0001. n=3 independent replicates. (F) Schematic diagram illustrating the p-IRF3/IRF7-dependent positive feedback loop downstream of the cGAS-STING pathway activates the RNA-sensing pathway, resulting in increased expression of OAS and IFIT family genes, followed by RNA degradation and translation inhibition.

Both mRNA abundance and translation efficiency are critical determinants of transgene expression. We examined whether the RNA-sensing pathway plays a role in suppressing transgene expression by focusing on gene families linked to RNA degradation and translational repression among the highly expressed IIR genes in cells with low transfection efficiency. These gene families include 2',5'-oligoadenylate synthetase (OAS) and interferon-induced proteins with tetratricopeptide repeats (IFITs). Once activated by exogenous RNA, the OAS family initiates the synthesis of 2',5'- oligoadenylates, which in turn activate RNaseL to degrade the exogenous RNA 46. The IFIT family comprises proteins that recognize and bind to improperly modified foreign RNAs to effectively block protein translation 47. Notably, all members of these families were consistently upregulated when the cells were transfected with plasmids (**Figure 2D, Figure 4A**, **Figure S10A**).

Next, we investigated whether these OAS and IFIT genes were indeed downstream of the cGAS-STING and RNA-sensing pathways. To this end, we quantified the expression levels of the OAS1/2/3/L and IFIT1/2/3 genes in NCM460 and L02 cells transfected with pcDNA3.1-GFP while silencing key components of the DNA- and RNA-sensing pathways. The knockdown efficiency of siRNAs targeting cGAS, STING, IRF3, IRF7, MDA5, and RIGI was validated (**Figures S11 and S12**). We detected a significant reduction in the expression of OAS1/2/3/L and IFIT1/2/3 when the DNA sensor STING or IRFs were depleted in cells. Similarly, the knockdown of the RNA sensors MDA5 and RIGI also led to significant decreases in OAS1/2/3/L and IFIT1/2/3 expression. When we performed a double knockdown to simultaneously deplete the factors involved in both pathways, a decrease in the transcription of the OAS1/2/3/L and IFIT1/2/3 genes was also detected. Notably, maximal effects were observed when both STING and MDA5 were targeted, revealing a synergistic role of DNA and RNA sensing in ISG activation and transgene silencing (**Figure 4B**, **Figure S10B**, **Figure S13A** and** B**).

After discovering that DNA and RNA sensing are key pathways that prevent cells from expressing transgenes, we were inspired to test whether knocking down key genes involved in DNA and RNA sensing could improve the efficiency of transgene expression. This approach could be developed into a generalized protocol for transgene expression that would significantly enhance biomedical applications. We performed flow cytometry to quantify the percentage of GFP-positive NCM460 and L02 cells following transfection with pcDNA3.1-GFP, which both exhibited low baseline transfection efficiency, after the DNA- and RNA-sensing genes described above were silenced to assess the applicability of this protocol (**Figure 4C** and **Figure S13C**). The results revealed a marked increase in the proportion of GFP-positive cells upon the knockdown of these immune-sensing components, with the most pronounced increase observed in the STING and MDA5 double-knockdown group (**Figure 4D** and **Figure S13D**). In addition to quantifying the proportion of transfected cells, we further evaluated overall transgene expression levels using qPCR, Western blot, and dual-luciferase reporter assays. These results reinforced our finding that the DNA- and RNA-sensing pathways suppress transgene expression (**Figure 4E**, **Figure S13E**, and **Figure S14**). Consistent with the observed reductions in OAS1/2/3/L and IFIT1/2/3 expression (**Figure 4B**, **Figure S10B**), the greatest increase in transgene expression occurred upon double knockdown of MDA5 and STING. We also confirmed that this effect was consistent across different transfection reagents and protocols (see **Figure S15**). We applied two different siRNAs targeting STING and MDA5 to exclude the possibility of off-target effects of the siRNAs, and, as expected, we steadily observed decreased expression of IIR genes and increased expression of GFP (**Figure S16** and **Table S3**).

To this end, we identified that the simultaneous targeting of two key factors, STING and MDA5, produced the most promising increase in transfection efficiency. Given that MDA5 is regulated by IRF3 (**Figure S8A** and** C**), we wondered whether cotargeting STING and IRF3 could produce a comparable increase in transfection efficiency in NCM460 and L02 cells. However, this combination did not yield the same level of improvement as STING and MDA5 double knockdown did (**Figure S17** and **Figure S13C-D**). We reasoned that this outcome might be due to the insufficient suppression of MDA5 following IRF3 depletion. As shown in **Figure S8A**, when we depleted IRF3, the mRNA of MDA5 remained up to 50%. IRF3 might be only one of the multiple factors contributing to the activation of MDA5. The residual MDA5 in IRF3-depleted cells could have already provided adequate RNA sensing for the cells to suppress transgene expression. Therefore, an exploration of additional regulators of MDA5 is needed to fully understand the cellular response to plasmid transfection.

Finally, we propose a model in which the uptake of foreign DNA leads to early activation of cGAS-STING-IRF3, followed by the induction of IRF7, which in turn drives the expression of RNA sensors such as MDA5 and RIGI. These RNA sensors further regulate IRF3 and IRF7, establishing a positive feedback loop and enhancing the immune response. Notably, the binding of p-IRF3/IRF7 to the promoters of IIR genes facilitates epigenetic remodeling at these loci, resulting in long-term cellular memory that enables a more rapid response to subsequent plasmid transfection. Following the activation of DNA- and RNA-sensing pathways, a cascade of downstream events is triggered, including the activation of the OAS and IFIT gene families, which promote mRNA degradation and inhibit protein translation. These processes effectively enhance the host cell's innate immune response and limit the expression of foreign DNA (**Figure 4F**).

## Discussion

The introduction of foreign genes into mammalian cells via transfection has broad applications in biological research, with transfection efficiency being a critical determinant of experimental success. HEK293T cells are well known for their exceptionally high transfection efficiency. However, many other cell types exhibit substantially lower efficiency. In this study, we observed that, compared with HEK293T cells, four other cell lines (HCT116, HeLa, L02, and NCM460) presented a shared cellular immune response to both linear and circular plasmid DNA. Notably, linearized plasmids elicited a markedly stronger immune response in these cells. In our transcriptomic analysis, the vast majority (i.e., >90%) of the cellular genes exhibited no significant changes in expression. However, a subset of genes showed significant activation. This observation suggests that, in addition to the expression of exogenous genes, plasmid DNA transfected into cells also triggers the activation of cellular genes, particularly in HeLa cells that presented more than 700 upregulated genes, many of which are associated with the innate immune response. This finding also raises concerns about the inevitable cellular response in biomedical research using plasmid transfection. Further investigation into the epigenetic mechanisms in cells transfected with foreign DNA revealed that the cGAS-STING pathway, which includes IRF3 and IRF7 within this pathway, along with their downstream RNA sensor genes (such as MDA5 and RIGI), was activated. Additionally, we confirmed that silencing cGAS, STING, IRF3, IRF7, MDA5, and RIGI significantly reduced the cellular expression levels of the OAS and IFIT gene families, which in turn mitigated mRNA degradation and translation inhibition and thus improved the transfection efficiency. This effect was especially notable in the STING and MDA5 double-knockdown groups. This study revealed that modulating the cGAS-STING and RNA-sensing pathways could increase transfection efficiency in cell types with lower baseline transfection rates.

In an earlier study 48, cells displaying a low transfection efficiency, such as PC-3 cells, presented significant activation of cytokine-stimulated genes, similar to what we observed here. However, the role of RNA sensing in affecting transfection efficiency was not analyzed. When we revisited their data, we found that RNA sensors (MDA5 and RIGI) were indeed activated in transfected PC-3 cells, supporting our findings that both DNA and RNA sensors are important for suppressing transfected DNA-mediated gene expression and that double knockdown of both types of nucleic acid sensors could maximize the transfection efficiency. In another study 49, plasmid transfection did not activate the RIGI/MAVS pathway, as evidenced by the unchanged GFP expression in both MAVS-deficient L929 cells and primary MEFs. Here, we showed that the key RNA sensors MDA5 and RIGI were activated by plasmid DNA transfection but not their potential downstream target MAVS, suggesting that alternative downstream signals of MDA5 and RIGI play a role in activating mRNA degradation. Additional efforts are needed to delineate the array of molecules involved in this pathway.

Lehner et al. intriguingly observed that transfection of circular plasmid DNA led to higher GFP expression in HeLa cells than did the transfection of linearized plasmid DNA, regardless of whether Lipofectamine or polyethyleneimine was used as the transfection reagent 50. However, the mechanistic basis for this difference remains unclear. In our study, we showed that the differential immune responses triggered by the transfection of distinct plasmid forms accounted for the observed variation in transfection efficiency. Notably, linear plasmids were significantly more effective at inducing innate immune responses (**Figure 2F**). This phenomenon was particularly evident in HEK293T cells, where compared with circular plasmids, the transfection of linear DNA resulted in a nearly fivefold increase in the activation of the RNA sensors MDA5 and RIGI.

HEK293T cells were originally immortalized through the expression of adenoviral E1A and E1B oncogenes and were later further engineered to express the SV40 large T antigen 30, 51. These viral oncogenes have been reported to suppress the cGAS-STING pathway 30, which is a key component of the cytosolic DNA-sensing machinery and plays a critical role in innate immune activation and downstream signaling. Such suppression may weaken cellular responses to exogenous DNA, thereby contributing to the high transfection efficiency commonly observed in HEK293T cells. In addition to HEK293T cells, we found that cGAS was not expressed in HCT116 cells, consistent with previous reports 32. Despite the absence of a DNA sensor, HCT116 cells exhibited a robust innate immune response and low transfection efficiency (**Figures 1** and** 2**). Notably, HCT116 cells expressed significantly higher levels of RNA sensors, particularly MDA5, which was approximately 11-fold more abundant than in HEK293T cells. This marked difference in RNA sensor expression may help explain the disparity in transfection efficiency between the two cell lines, underscoring the pivotal role of RNA sensors in initiating immune responses during plasmid DNA transfection.

In contrast, HeLa cells are well known for their high transfection efficiency and satisfactory ectopic expression. However, more IIR genes are activated in these cells than in cell lines with very low transfection efficiency, such as NCM460 cells (**Figures 1** and **2**). This phenomenon may be closely associated with the high metabolic activity characteristic of cancer cells. Cancer cells typically undergo metabolic reprogramming, which includes increased glycolysis (even under aerobic conditions, known as the Warburg effect), increased nucleotide and protein biosynthesis, and increased mitochondrial function, to support rapid cell proliferation 52, 53. For example, HeLa cells constitutively express the HPV18 oncogenic proteins E6 and E7, which are considered key drivers of their high proliferative capacity and metabolic reprogramming 54. These metabolic features may act synergistically to create a more favorable intracellular environment for the transcription and translation of plasmid DNA. For example, active biosynthetic metabolism can increase transcriptional and translational activities, thereby promoting increased levels of exogenous gene expression. Consequently, despite the activation of strong immune responses following plasmid transfection, the high metabolic state of cancer cells may, to some extent, buffer the suppressive effects of immune signaling on transgene expression, ultimately resulting in increased transfection efficiency.

The DExD/H-box (DDX) RNA helicase family is known for its role in unwinding RNA internal structures 55-57. In addition to RIGI, which is encoded by the RIGI gene, we have also discovered that DDX60 can be significantly upregulated in four cell lines with low transfection efficiency (**Table S1**), which is mediated by the binding of IRF7 and p-IRF3 (**Figure 3F**). Previous studies have shown that DDX60 colocalizes with RIGI and MDA5, enhancing their regulation of type I interferon gene expression 58, and its knockdown significantly weakens the activation of the IFN-β promoter in response to dsRNA 59. Intriguingly, knocking down DDX60 alone failed to significantly increase our cell transfection efficiency (data not shown), suggesting that DDX60 may not be the primary RNA sensor sentinel playing a major role in the process of plasmid transfection.

Recent work has shown that plasmid DNA transfection activates innate immune responses through two distinct pathways: PKR-mediated stress granule (SG) formation and the cGAS-STING axis 32. The study showed that the transcription of plasmid-derived RNA leads to the formation of dsRNA, which activates PKR, resulting in eIF2α phosphorylation and global translational suppression via SG assembly. Knockdown of PKR significantly increased transgene expression, suggesting that PKR-mediated signaling can restrict transgene expression in HeLa cells. Nevertheless, our data also revealed PKR activation in HEK293T cells, which presented the highest transfection efficiency (**Figure S18**), indicating that PKR may not be the sole factor affecting transfection efficiency. These findings point to the existence of additional, cell-type-specific factors that modulate the transfection response beyond PKR-mediated effects.

## Supplementary Material

Supplementary figures and tables.
