# Supplementary material for: Unlocking the Puzzle of Mammalian Transfection: The Role of the RNA-sensing-Mediated Interferon Response in the Cellular Defense Against Foreign DNA Intrusion
Source: Int J Biol Sci. 2025 Jun 9;21(9):3886–900. doi: 10.7150/ijbs.107510 (PMC12210383; doi:10.7150/ijbs.107510)
Supplement: Supplementary file 1 — Supplementary figures and tables. [file ijbsv21p3886s1.pdf]

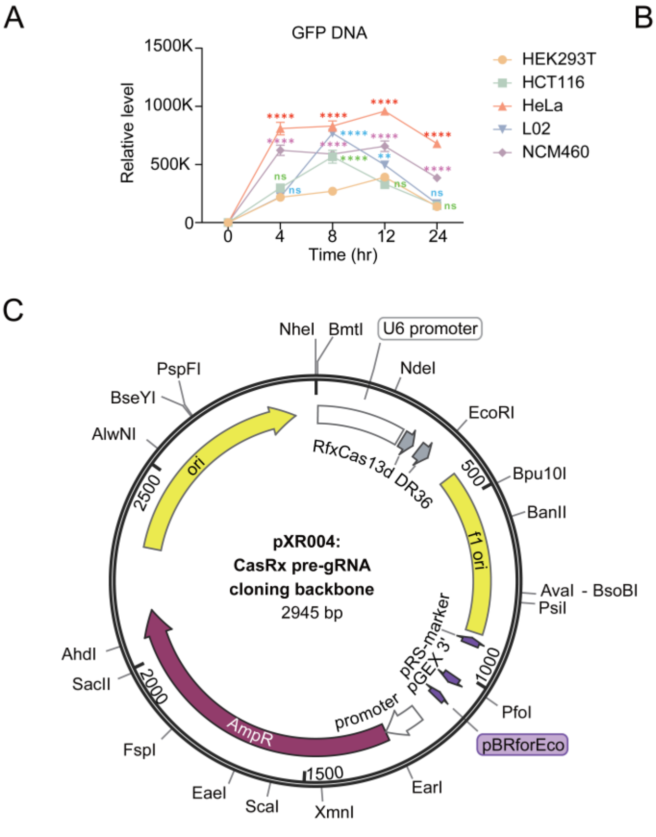

Figure S1. Comparison of plasmid transfection and transgene expression across cell lines.

qPCR analysis of pcDNA3.1-GFP plasmid DNA **(A)** and EGFP mRNA (**B**) levels normalized to those of GAPDH at the indicated time points (≤24 h) in transfected HEK293T, HCT116, HeLa, L02, and NCM460 cells. Statistical P values were calculated using Dunnett's test following ANOVA, with HEK293T cells transfected with pcDNA3.1-GFP serving as the control group. *P < 0.05, **P < 0.01, ***P < 0.001 and ****P < 0.0001. ns, not statistically significant. n=3 independent replicates. **(C)** Maps of the pre-gRNA and pcDNA3.1-neo plasmids.

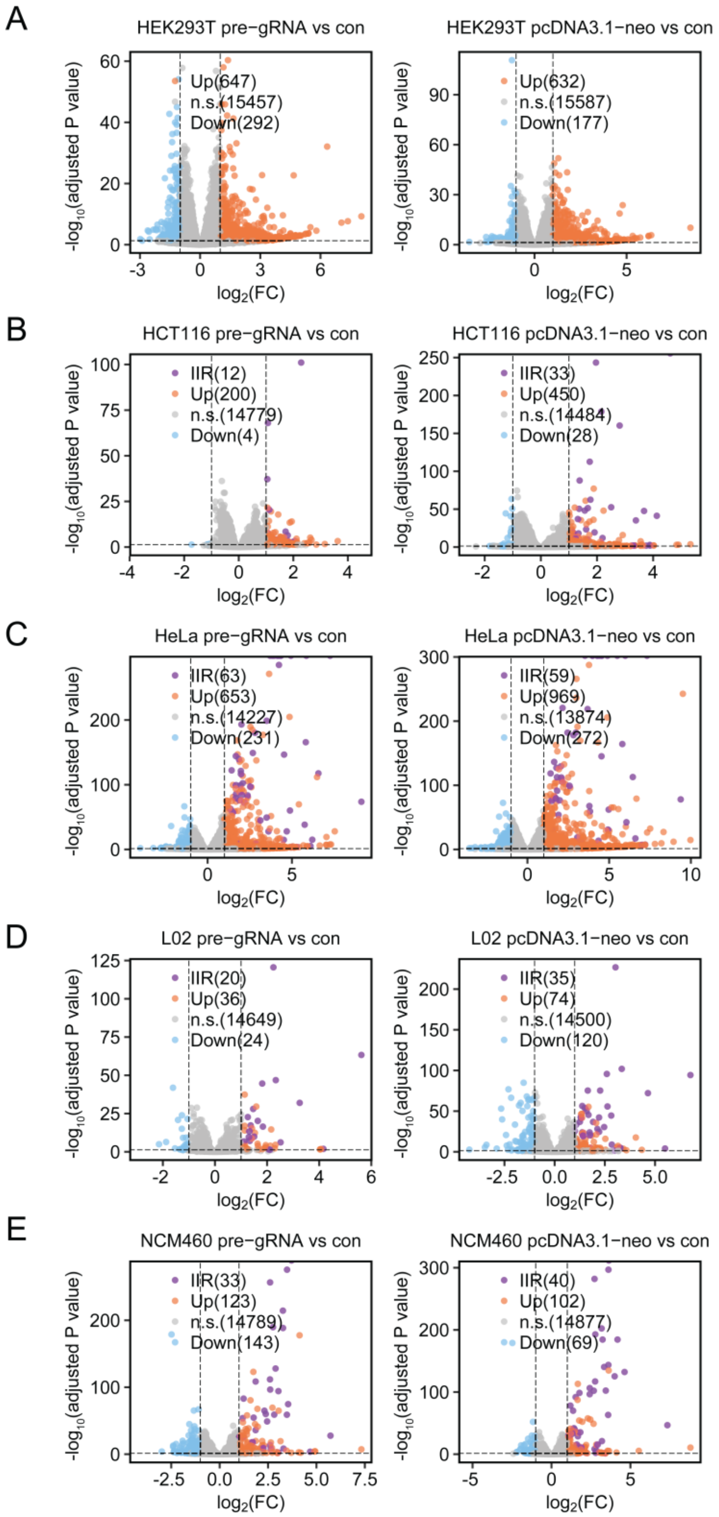

Figure S2. Gene expression varies across cell types after circular plasmid transfection.

Volcano plots of DEGs identified in the HEK293T **(A)**, HCT116 **(B)**, HeLa **(C)**, L02 **(D)**, and NCM460 **(E)** cell lines transfected with the pre-gRNA or pcDNA3.1-neo plasmid compared with the controls. IIR: innate immune response genes related to the RTV BP term, "Up", upregulated (fold change ≥ 2, adjusted p value ≤ 0.05), "n.s. ", not significant, "Down", downregulated (fold change ≤ -2, adjusted p value ≤ 0.05), and "con", control. n=3 independent replicates.

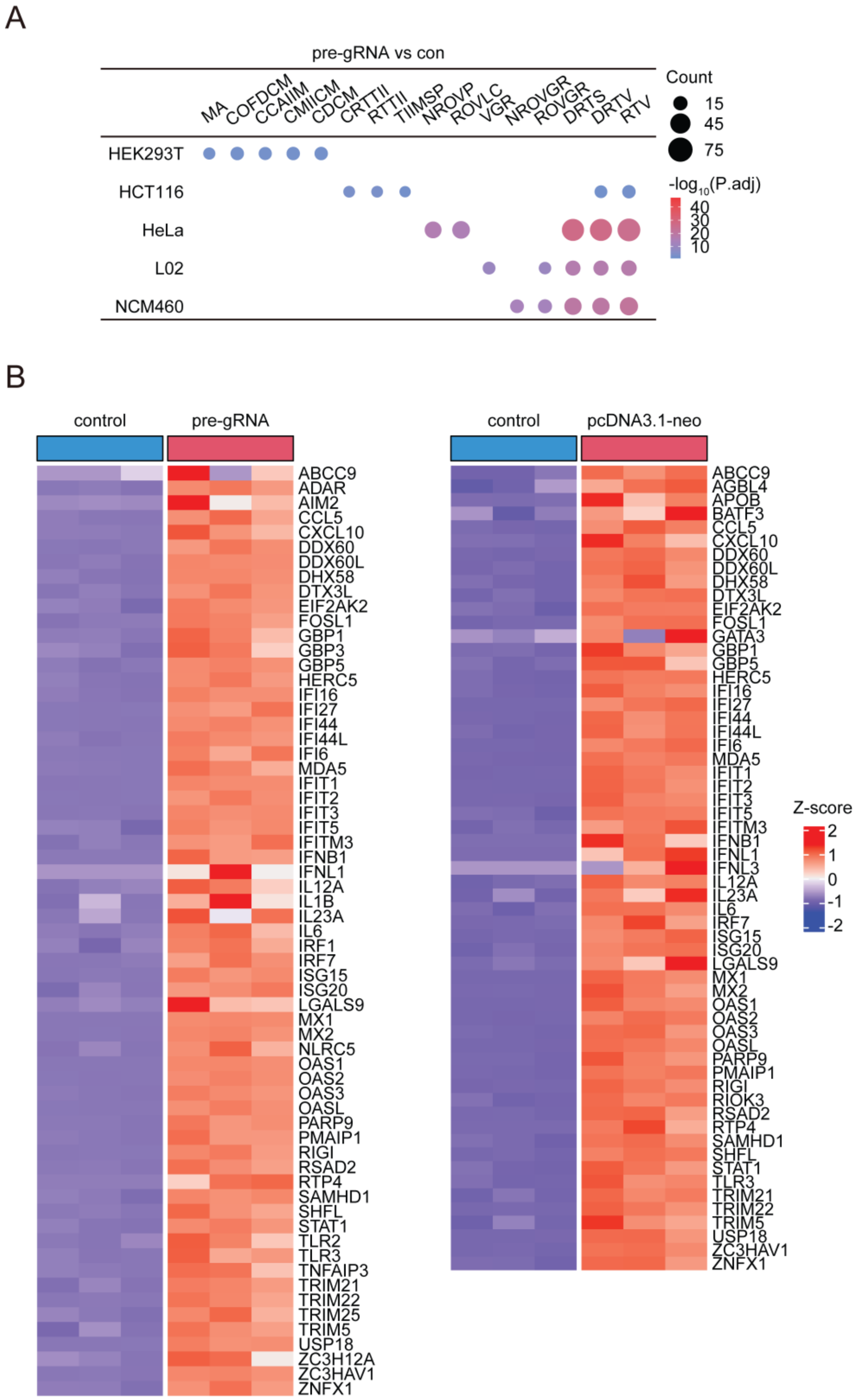

Figure S3. Circular plasmid transfection induces the broad upregulation of IIR genes in HeLa cells.

(A) Dot plots displaying the top five most significantly enriched biological process GO terms among the upregulated DEGs in each cell line following pre-gRNA plasmid transfection versus controls. MA: myofibril assembly; COFDCM: cilium or flagellum-dependent cell motility; CCAIIM: cellular component assembly involved in morphogenesis; CMIICM: cilium movement involved in cell motility; CDCM: cilium-dependent cell motility; CRTTII: cellular response to type I interferon; RTTII: response to type I interferon; TIIMSP: type I

interferon-mediated signaling pathway; NROVP: negative regulation of viral process; ROVLC: regulation of the viral life cycle; VGR: viral genome replication; NROVGR: negative regulation of viral genome replication; ROVGR: regulation of viral genome replication; DRTS: defense response to symbionts; DRTV: defense response to virus; RTV: response to virus. P.adj: adjusted p value. n=3 independent replicates. **(B)** Heatmaps displaying the differential expression of IIR genes in HeLa cells transfected with pre-gRNA (left panel) or pcDNA3.1-neo (right panel) plasmids compared with the controls. n=3 independent replicates.

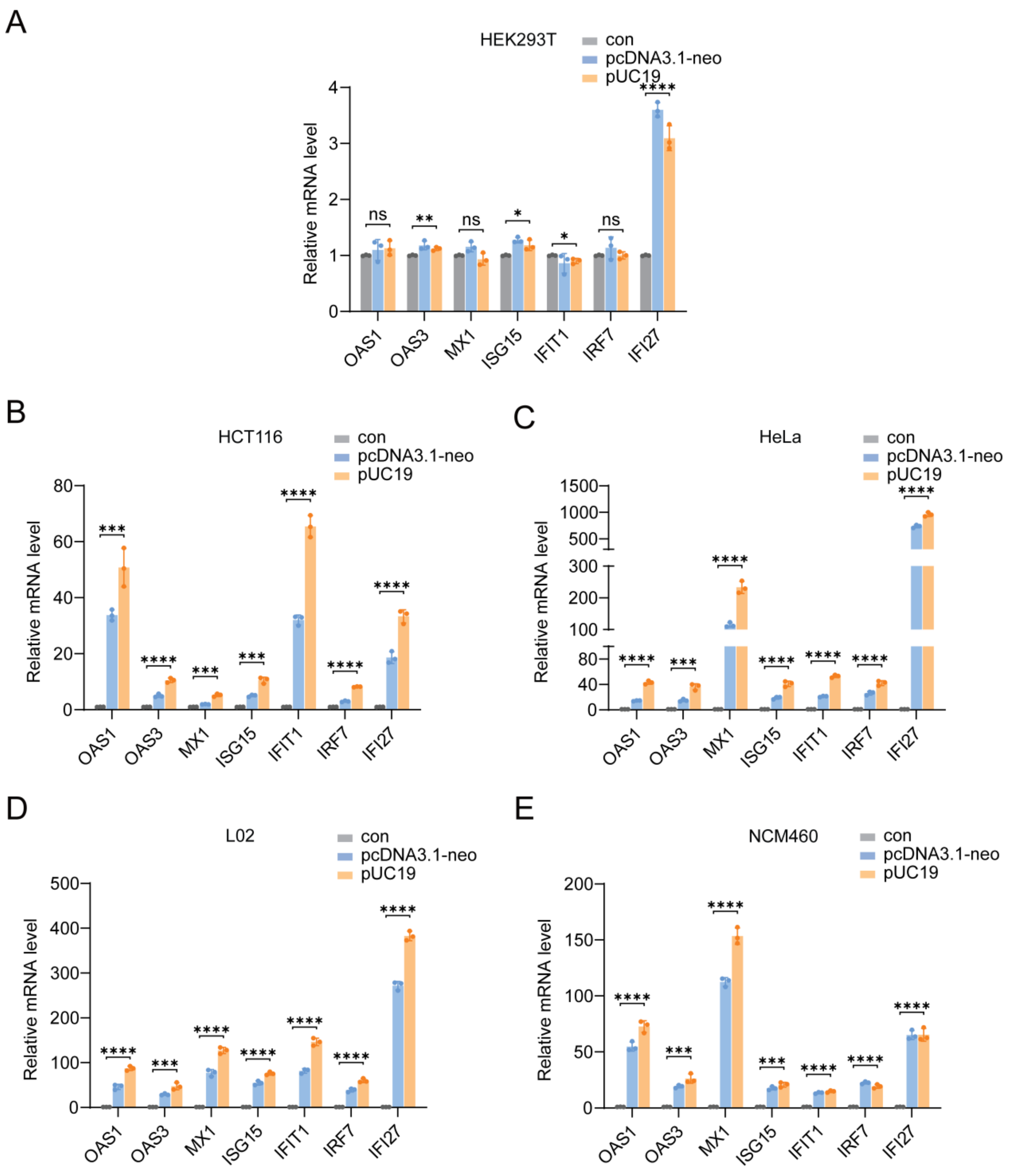

Figure S4. Identification of IIR genes activated by the pUC19 plasmid.

qPCR results at 24 h reveal the relative mRNA expression levels of 7 IIR genes in HEK293T **(A)**, HCT116 **(B)**, HeLa **(C)**, L02 **(D)**, and NCM460 **(E)** cells treated with either transfection reagent, pcDNA3.1-neo, or pUC19 plasmids. Con: control. Statistical comparisons between the pUC19-treated groups and the control groups were performed using two-tailed Student's t tests. *P < 0.05, **P <0.01, ***P < 0.001 and ****P < 0.0001. n=3 independent replicates.

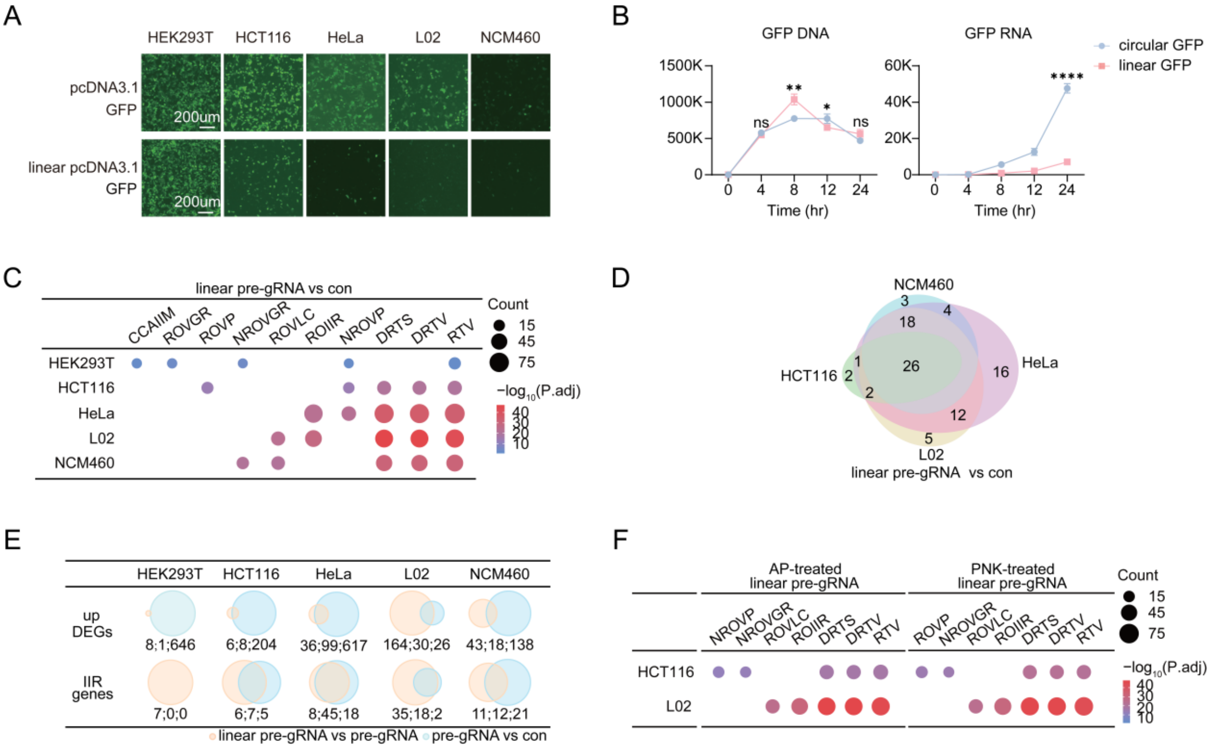

Figure S5. The linear pre-gRNA plasmid significantly activates IIR genes.

(A) GFP fluorescence in HEK293T, HCT116, HeLa, L02, and NCM460 cells transfected with either circular or linear pcDNA3.1-GFP plasmids. **(B)** qPCR analysis of pcDNA3.1-GFP plasmid DNA and EGFP mRNA levels normalized to those of GAPDH at the indicated time points (≤24 h) in transfected NCM460 cells. Statistical significance was determined using two-tailed Student's t tests. *P < 0.05, **P < 0.01, ***P < 0.001 and ****P < 0.0001. ns, not statistically significant. n=3 independent replicates. **(C)** Dot plots displaying the top five most significantly enriched biological process GO terms among the upregulated DEGs in each cell line following linear pre-gRNA plasmid transfection versus controls. CCAIIM: cellular component assembly involved in morphogenesis; ROVGR: regulation of viral genome replication; ROVP: regulation of viral processes; NROVGR: negative regulation of viral genome replication; ROVLC: regulation of the viral life cycle; ROIIR: regulation of the innate immune response; NROVP: negative regulation of viral processes; DRTS: defense response to symbionts; DRTV: defense response to virus; RTV: response to virus. P.adj: adjusted p value. n=3 independent replicates. **(D)** Venn diagram of IIR genes activated by the linear pre-gRNA plasmid compared with the transfection reagent alone across the HCT116, HeLa, L02, and NCM460 cell lines. Con: control. n=3 independent replicates. **(E)** Venn diagrams illustrating the upregulated DEGs and IIR genes between linear pre-gRNA and pre-gRNA plasmid transfections (yellow) and between pre-gRNA plasmid transfections and controls (blue). Con: control. The numerical values corresponding to each Venn diagram region are annotated below and separated by colons. n=3 independent replicates. **(F)** Dot plots displaying the top five enriched GO biological process terms among the upregulated DEGs in HCT116 and L02 cells transfected with linear pre-gRNA plasmids treated with alkaline phosphatase (left panel) or polynucleotide kinase (right panel) compared with the controls. NROVP: negative regulation of the viral process; NROVGR: negative regulation of viral genome replication; ROVLC: regulation of the viral life cycle; ROIIR: regulation of the innate immune response; DRTS: defense response to symbionts; DRTV: defense response to virus; RTV: response to virus. AP: alkaline phosphatase. PNK: polynucleotide kinase. P.adj: adjusted p value. n=3 independent replicates.

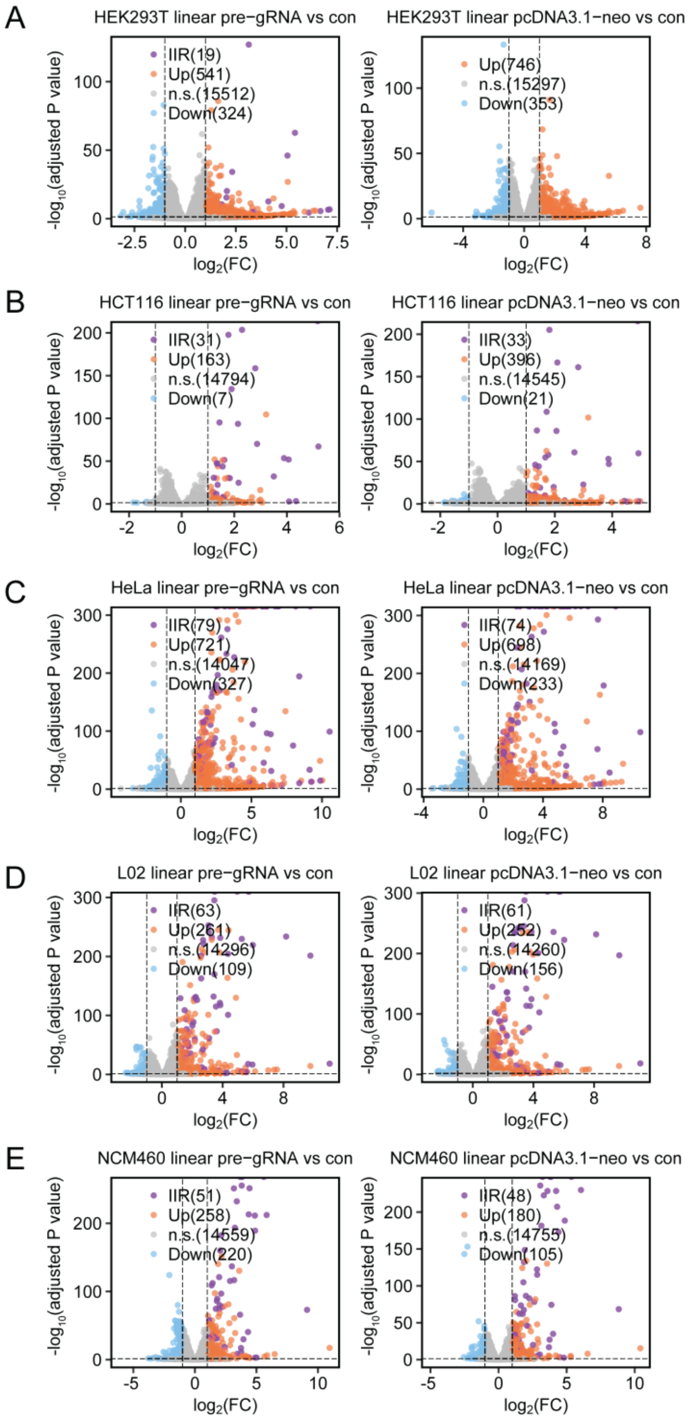

Figure S6. Gene expression varies across cell types after linear plasmid transfection.

Volcano plots of DEGs identified in the HEK293T **(A)**, HCT116 **(B)**, HeLa **(C)**, L02 **(D)**, and NCM460 **(E)** cell lines transfected with linear pre-gRNA or linear pcDNA3.1-neo plasmid compared with the controls. IIR: innate immune response genes related to the RTV BP term, "Up", upregulated (fold change ≥ 2, adjusted p value ≤ 0.05), "n.s.", not significant, "Down", downregulated (fold change ≤ -2, adjusted p value ≤ 0.05), and "con", control. n=3 independent replicates.

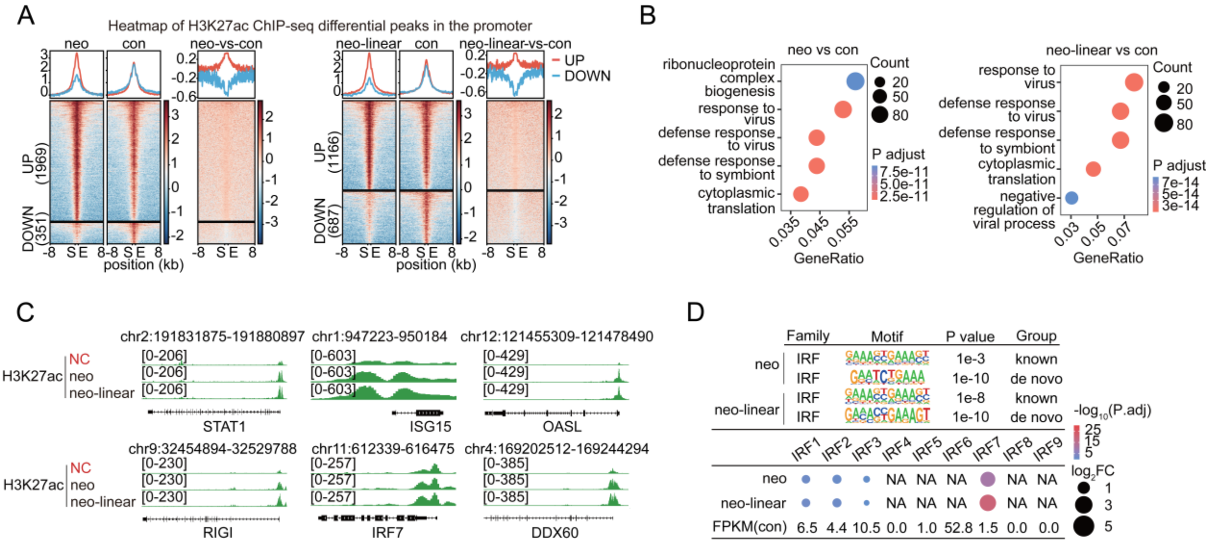

Figure S7. Epigenetic changes in NCM460 cells after transfection with circular and linear pcDNA3.1-neo plasmids.

(A) Averaged profiles (top panels) and heatmaps (bottom panels) of H3K27ac ChIP-seq signals within the ±8 kb regions flanking the differential peaks on the promoters identified in NCM460 cells transfected with circular or linear pcDNA3.1-neo plasmids compared with the control. The counts of upregulated and downregulated peaks are displayed on the left side of the heatmaps. Con: control. S: peak start site. E: peak end site. UP: upregulated peaks. DOWN: downregulated peaks. neo: pcDNA3.1-neo, neo-linear: linear pcDNA3.1-neo. n=3 independent replicates. **(B)** GO results for genes with active promoter regions containing upregulated differentially abundant ChIP-seq peaks in NCM460 cells following transfection with pcDNA3.1-neo (left panel) or linear pcDNA3.1-neo (right panel) plasmids compared with the control groups. Con: control. P adjust: adjusted p value. neo: pcDNA3.1-neo, neo-linear: linear pcDNA3.1-neo. n=3 independent replicates. **(C)** H3K27ac ChIP-seq tracks for the STAT1, ISG15, OASL, RIGI, IRF7, and DDX60 genes in NCM460 cells treated with only the transfection reagent or transfected with pcDNA3.1-neo or linear pcDNA3.1-neo plasmids. NC: negative control, neo: pcDNA3.1-neo, neo-linear: linear pcDNA3.1-neo. n=3 independent replicates. **(D)** The most significant Homer motifs (top panel) of the IRF transcription factor family from the known or de novo motif enrichment results, along with dot plots (bottom panels) illustrating the differential expression levels of all genes in the IRF family in NCM460 cells transfected with pcDNA3.1-neo or linear pcDNA3.1-neo plasmids compared with the controls. Only data for genes with increased expression are shown. The average FPKM values of the IRF family genes in the control group are indicated at the bottom. P.adj: adjusted p value. FC: fold change. neo: pcDNA3.1-neo, neo-linear: linear pcDNA3.1-neo. n=3 independent replicates.

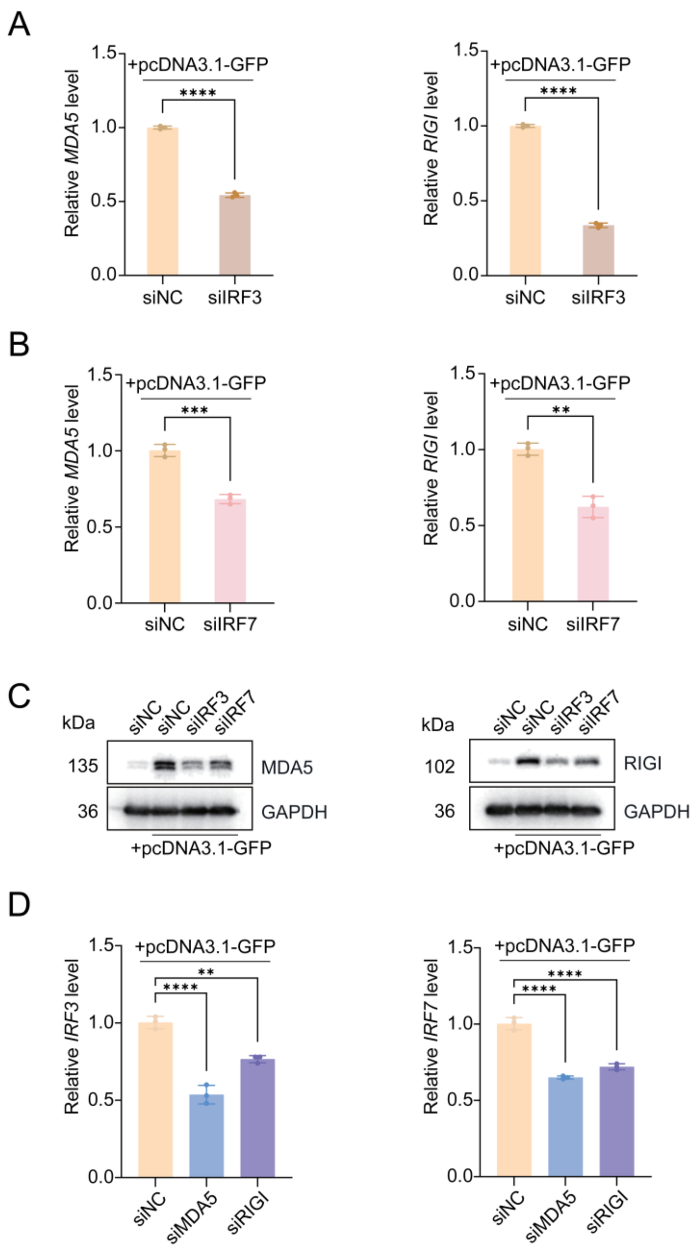

Figure S8. Evidence of mutual regulation between RNA sensors and IRF3/IRF7.

qPCR results at 24 h revealing the relative MDA5 and RIGI expression levels compared with those of the GAPDH gene after knocking down IRF3 **(A)** or IRF7 **(B)** in NCM460 cells transfected with pcDNA3.1-GFP plasmids. Statistical significance was determined using two-tailed Student's t tests. *P < 0.05, **P < 0.01, ***P < 0.001 and ****P < 0.0001. n=3 independent replicates. **(C)** Western blot analysis at 24 h showing the MDA5 and RIGI expression levels after knocking down IRF3 or IRF7 in NCM460 cells transfected with pcDNA3.1-GFP plasmids. **(D)** qPCR results at 24 h revealing the relative IRF3 and IRF7 expression levels compared with those of the GAPDH gene after knocking down MDA5 and RIGI in NCM460 cells transfected with pcDNA3.1-GFP plasmids. Statistical P values were calculated using Dunnett's test following ANOVA, with siNC cells transfected with pcDNA3.1-GFP used as the control group. siNC: negative control siRNA. *P < 0.05, **P < 0.01, ***P < 0.001 and ****P < 0.0001. n=3 independent replicates.

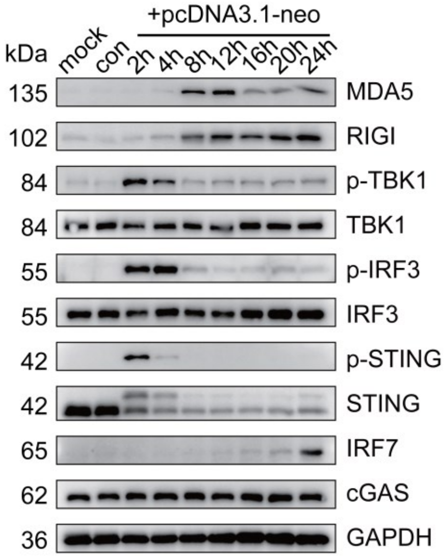

Figure S9. Western blotting of key proteins involved in the DNA- and RNA-sensing pathways.

Western blot assays examining MDA5, RIGI, p-TBK1, TBK1, p-IRF3, IRF3, p-STING, STING, IRF7, cGAS, and GAPDH levels in HeLa cells transfected with the pcDNA3.1-neo plasmid at various time points. Con: control groups treated with only the transfection reagent. mock: wild-type cells.

A

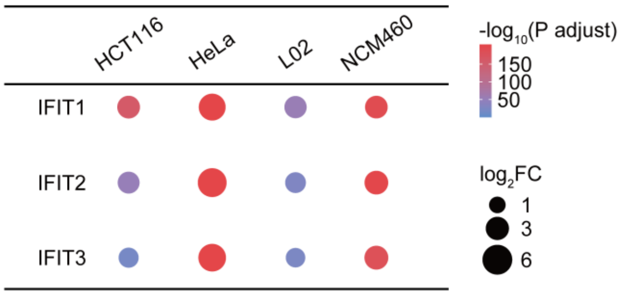

B

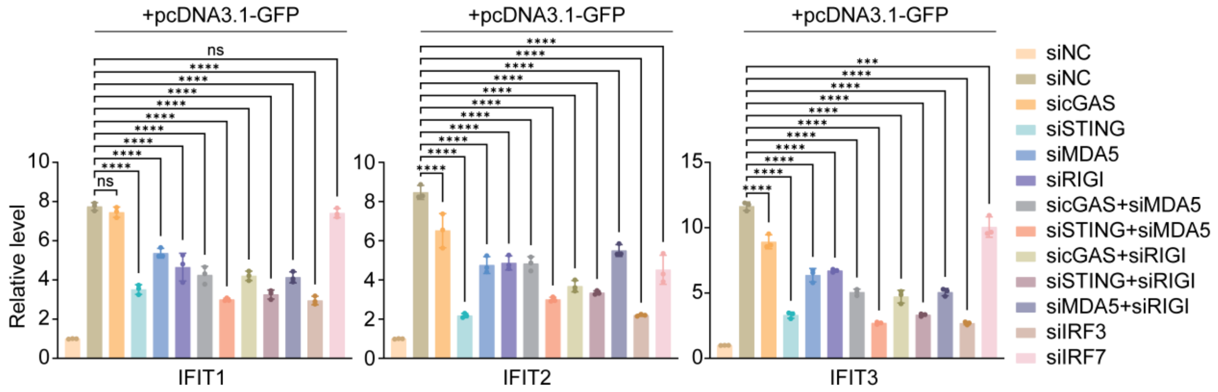

Figure S10. IFIT gene expression levels following the knockdown of key factors in the DNA- and RNA-sensing pathways.

(A) Dot plots showing the differential expression levels of IFIT1, IFIT2, and IFIT3 in HCT116, HeLa, L02, and NCM460 cells following transfection with the pcDNA3.1-neo plasmid. n=3 independent replicates. **(B)** qPCR results at 24 h revealing the IFIT1, IFIT2, and IFIT3 expression levels after the knockdown of cGAS, STING, MDA5, RIGI, IRF3, and IRF7, as well as the double knockdown of factors in the DNA- and RNA-sensing pathways in NCM460 cells transfected with pcDNA3.1-GFP plasmids. Statistical P values were calculated using Dunnett's test following ANOVA, with siNC cells transfected with pcDNA3.1-GFP used as the control group. siNC: negative control siRNA. *P < 0.05, **P <0.01, ***P < 0.001 and ****P < 0.0001. ns, not statistically significant. n=3 independent replicates.

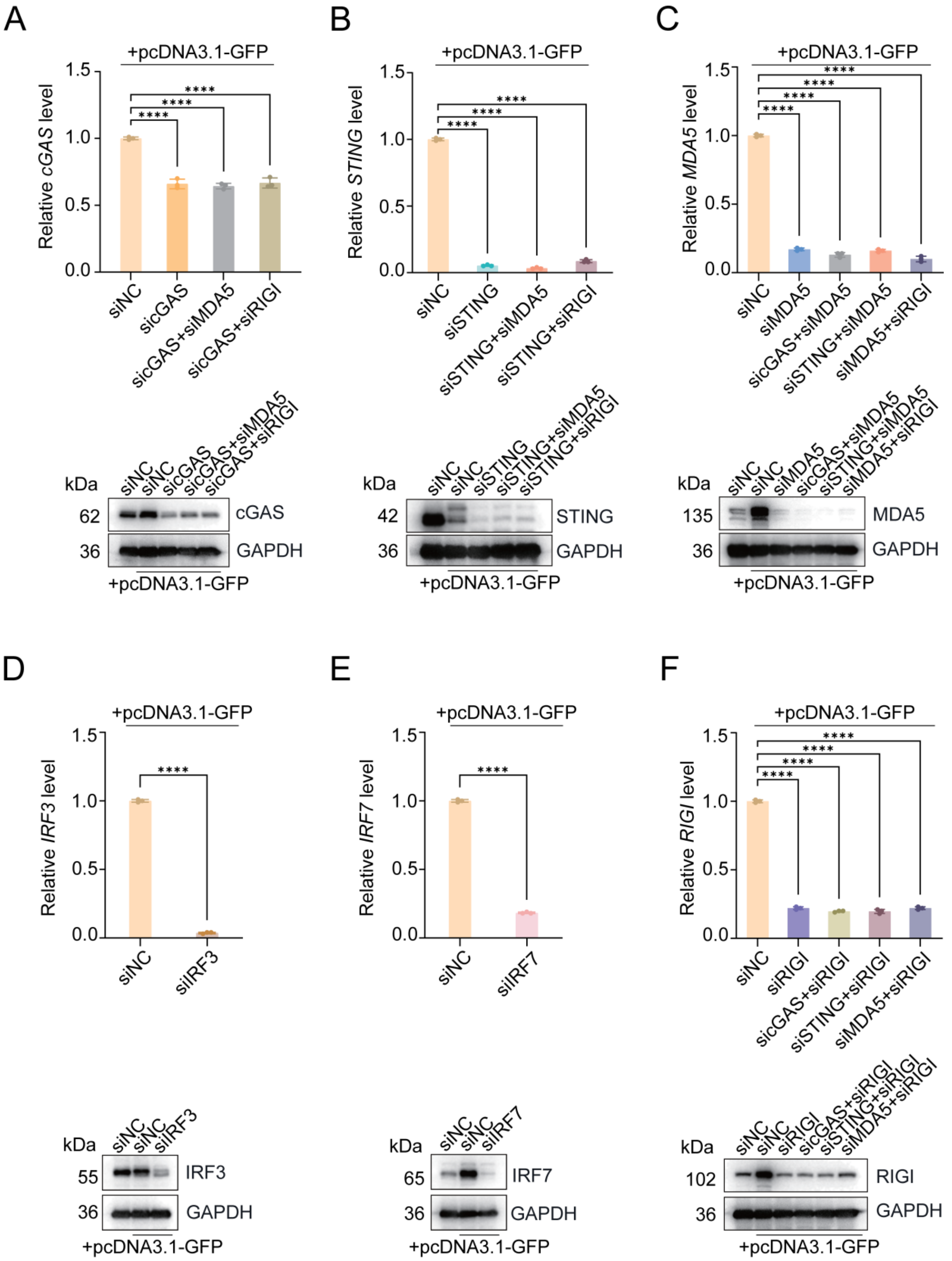

Figure S11. Efficiency of the knockdown of key factors in DNA- and RNA-sensing pathways in NCM460 cells.

qPCR results (upper panel) and Western blot results (lower panel) at 24 h showing the knockdown levels of siRNAs targeting cGAS **(A)**, STING **(B)**, MDA5 **(C)**, IRF3 **(D)**, IRF7 **(E)** and RIGI **(F)** in NCM460 cells transfected with pcDNA3.1-GFP plasmids. Statistical P values were calculated using Dunnett's test after ANOVA

for the data presented in (A), (B), (C), and (F) and via two-tailed Student's t test for the data presented in (D) and (E), with siNC cells transfected with pcDNA3.1-GFP used as the control group. siNC: negative control siRNA. *P < 0.05, **P < 0.01, ***P < 0.001 and ****P < 0.0001. n=3 independent replicates.

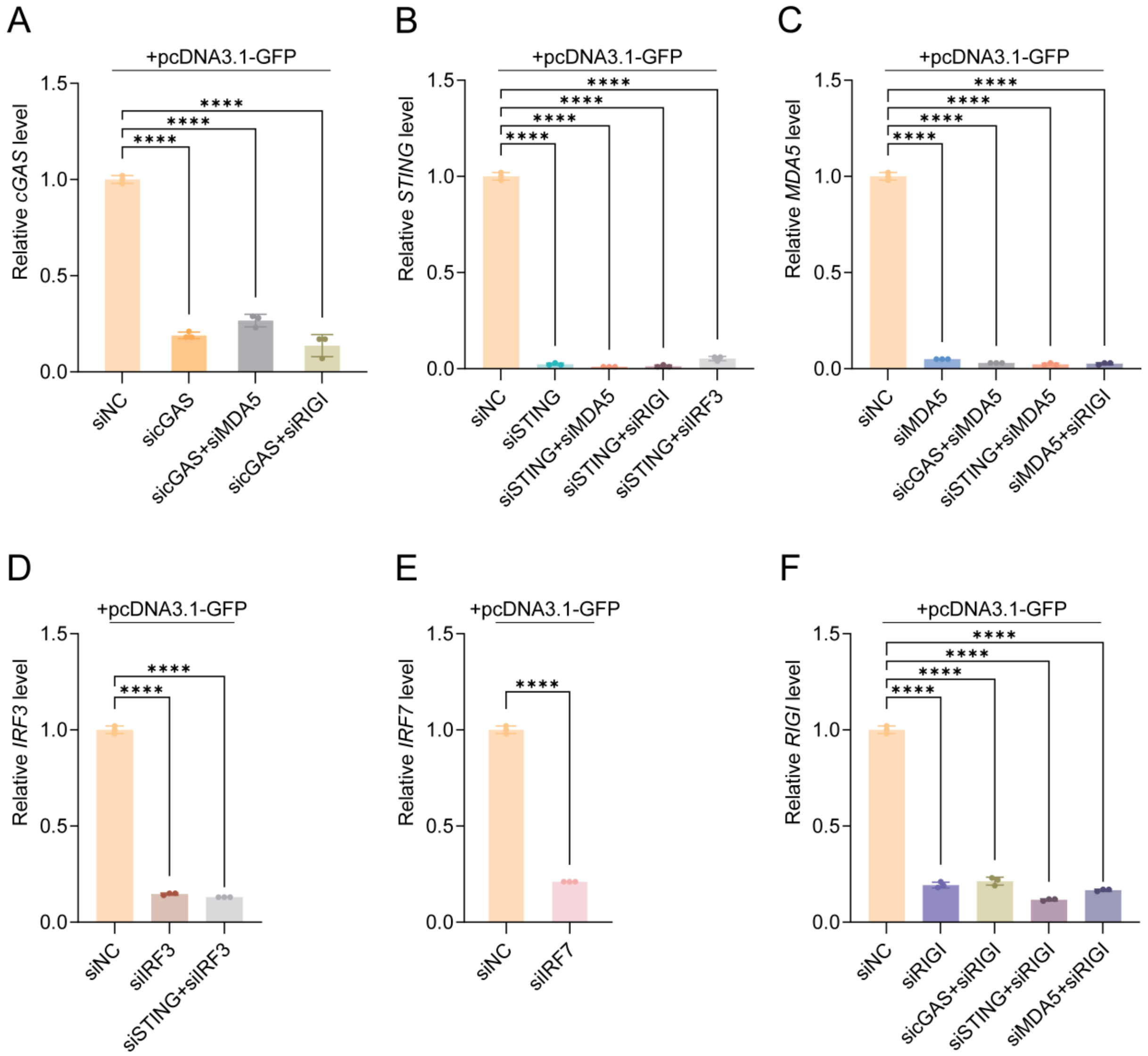

Figure S12. Efficiency of the knockdown of key factors in DNA- and RNA-sensing pathways in L02 cells. qPCR results at 24 h showing the knockdown levels of siRNAs targeting cGAS **(A)**, STING **(B)**, MDA5 **(C)**, IRF3 **(D)**, IRF7 **(E)**, and RIGI **(F)** in L02 cells transfected with pcDNA3.1-GFP plasmids. Statistical P values were calculated using Dunnett's test after ANOVA for the data presented in (A), (B), (C), (D), and (F) and via a two-tailed Student's t test for the data presented in (E), with siNC cells transfected with pcDNA3.1-GFP serving as the control group. siNC: negative control siRNA. *P < 0.05, **P < 0.01, ***P < 0.001 and ****P < 0.0001. n=3 independent replicates.

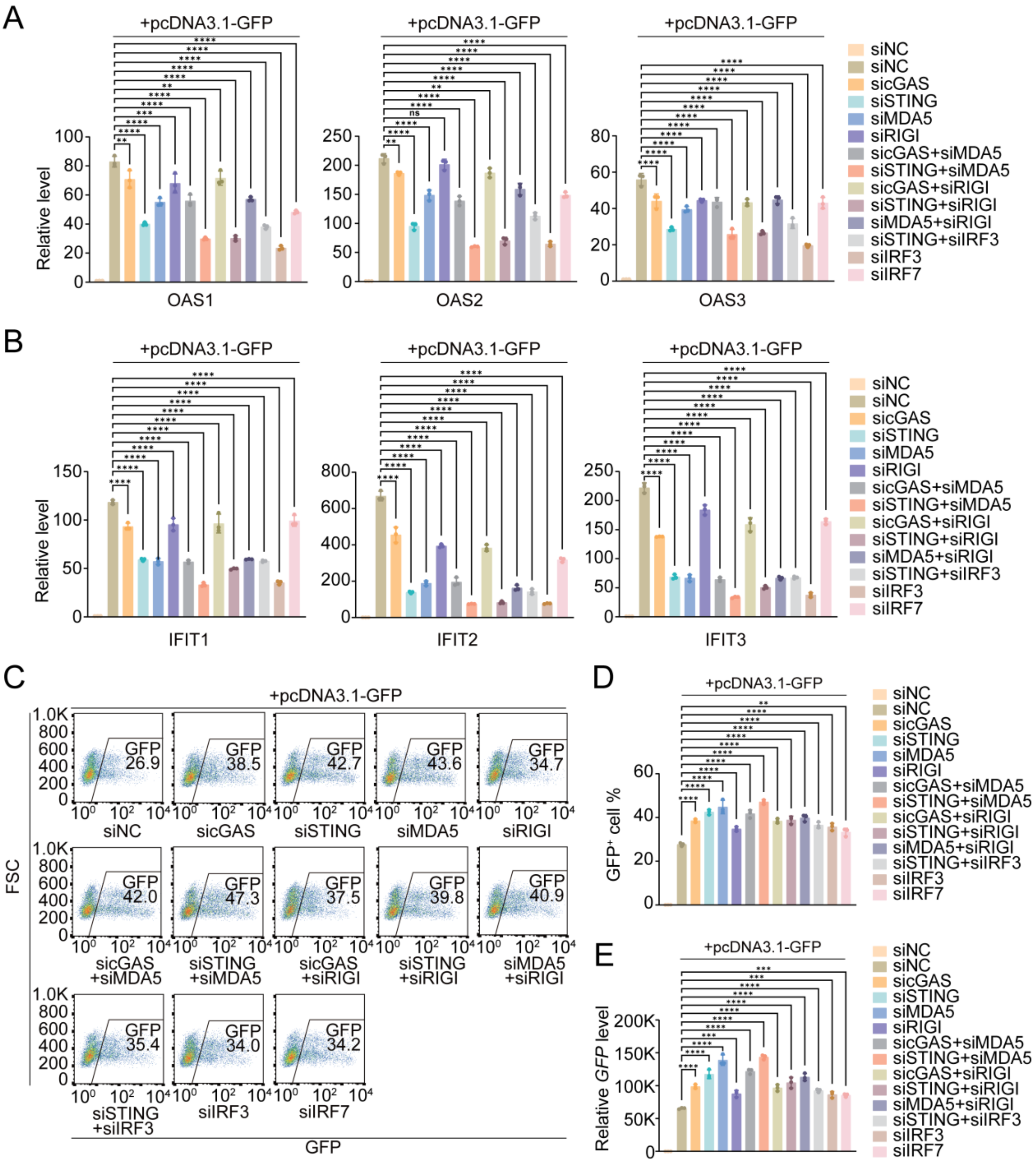

Figure S13. Knockdown of key DNA- and RNA-sensing factors increases the transfection efficiency in L02 cells.

qPCR results at 24 h revealing the relative OAS1/2/3 **(A)** and IFIT1/2/3 **(B)** expression levels compared with those of the GAPDH gene after the knockdown of the cGAS, STING, MDA5, RIGI, IRF3, and IRF7 genes, as well as the double knockdown of factors of the DNA- and RNA-sensing pathways in L02 cells transfected with pcDNA3.1-GFP plasmids. n=3 independent replicates. **(C)** Flow cytometry results of L02 cells transfected with pcDNA3.1-GFP plasmids following the knockdown of cGAS, STING, MDA5, RIGI, IRF3, and IRF7, as well as the double knockdown of factors of the DNA- and RNA-sensing pathways. NC: negative control. FSC: forward scatter. n=3 independent replicates. **(D)** GFP$^+$ cell ratio obtained from flow cytometry in (C). n=3 independent replicates. **(E)** qPCR results at 24 h revealing the relative GFP expression levels compared with those of the

GAPDH gene after the knockdown of cGAS, STING, MDA5, RIGI, IRF3, and IRF7, as well as the double knockdown of factors of the DNA- and RNA-sensing pathways in L02 cells transfected with pcDNA3.1-GFP plasmids. Statistical P values were calculated using Dunnett's test after ANOVA, with siNC cells transfected with pcDNA3.1-GFP used as the control group. siNC: negative control siRNA. *$P < 0.05$, **$P < 0.01$, ***$P < 0.001$, ****$P < 0.0001$, ns, not statistically significant. n=3 independent replicates.

A

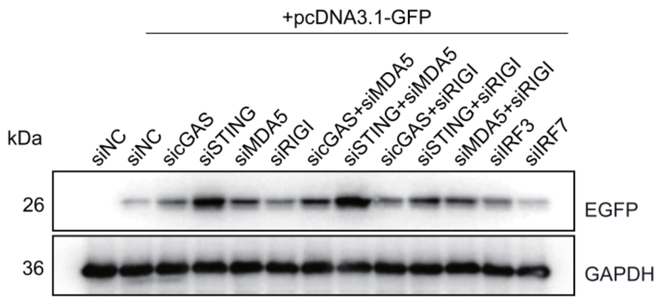

B

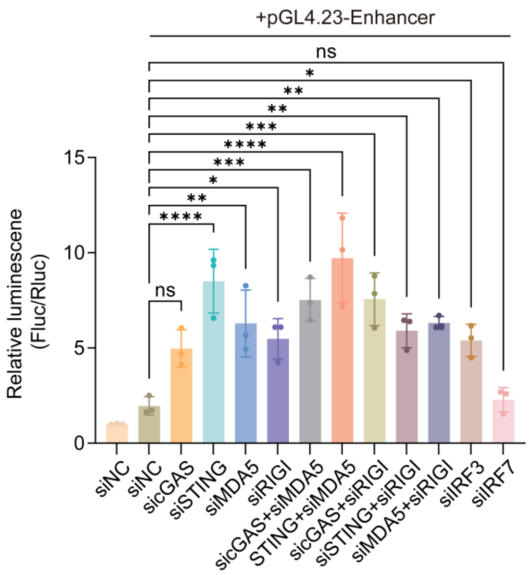

Figure S14. Increased transfection efficiency in NCM460 cells following the knockdown of DNA and RNA sensors.

(A) Western blot analysis at 24 h revealing GFP expression levels in NCM460 cells transfected with pcDNA3.1-GFP plasmids following the knockdown of cGAS, STING, MDA5, RIGI, IRF3, and IRF7, as well as the double knockdown of DNA- and RNA-sensing pathway factors. **(B)** Relative luminescence was analyzed using a dual luciferase reporter assay at 24 hours after the single-gene knockdown of cGAS, STING, MDA5, RIGI, IRF3, and IRF7 or double knockdown of both DNA- and RNA-sensing pathway factors in NCM460 cells transfected with pGL4.23-enhancer plasmids. Statistical significance was determined using Dunnett's test following ANOVA, with siNC cells transfected with pGL4.23-enhancer serving as the control group. siNC: negative control siRNA. *P < 0.05, **P < 0.01, ***P < 0.001 and ****P < 0.0001. ns, not statistically significant. Fluc: Firefly luciferase. Rluc: Renilla luciferase. n=3 independent replicates.

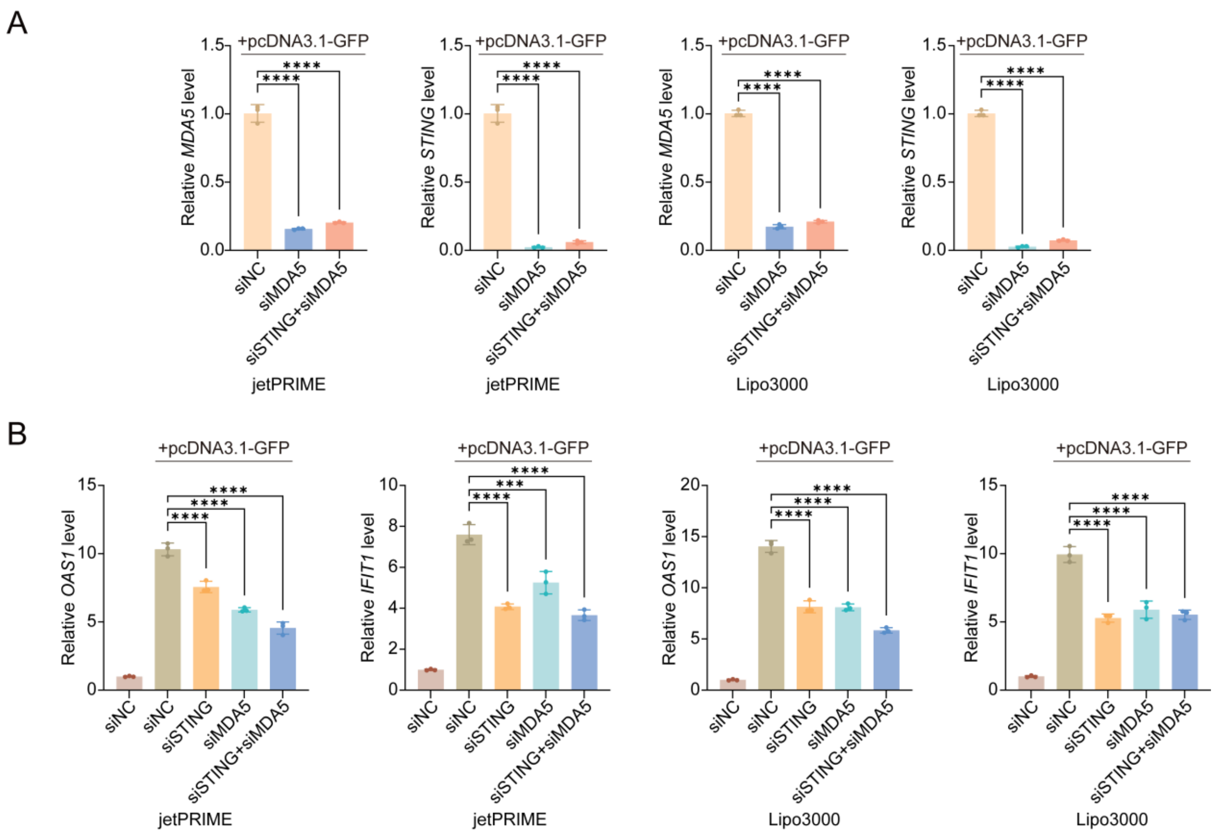

Figure S15. Knocking down STING and MDA5 reduces OAS1 and IFIT1 gene expression independent of the transfection reagent used.

(A) qPCR results at 24 h revealing the knockdown levels of siRNAs targeting MDA5 and STING in NCM460 cells transfected with pcDNA3.1-GFP using jetPRIME or Lipomaster 3000. Statistical significance was determined using Dunnett's test following ANOVA, with siNC cells transfected with pcDNA3.1-GFP used as the control group. siNC: negative control siRNA. *P < 0.05, **P < 0.01, ***P < 0.001, and ****P < 0.0001. Lipo3000: Lipomaster 3000; n=3 independent replicates. **(B)** qPCR results at 24 h revealing the relative OAS1 and IFIT1 expression levels compared with those of the GAPDH gene after the knockdown of STING or MDA5 in NCM460 cells transfected with pcDNA3.1-GFP plasmids using jetPRIME or Lipomaster 3000. Statistical significance was determined using Dunnett's test following ANOVA, with siNC cells transfected with pcDNA3.1-GFP used as the control group. siNC: negative control siRNA. *P < 0.05, **P < 0.01, ***P < 0.001, and ****P < 0.0001. Lipo3000: Lipomaster 3000; n=3 independent replicates.

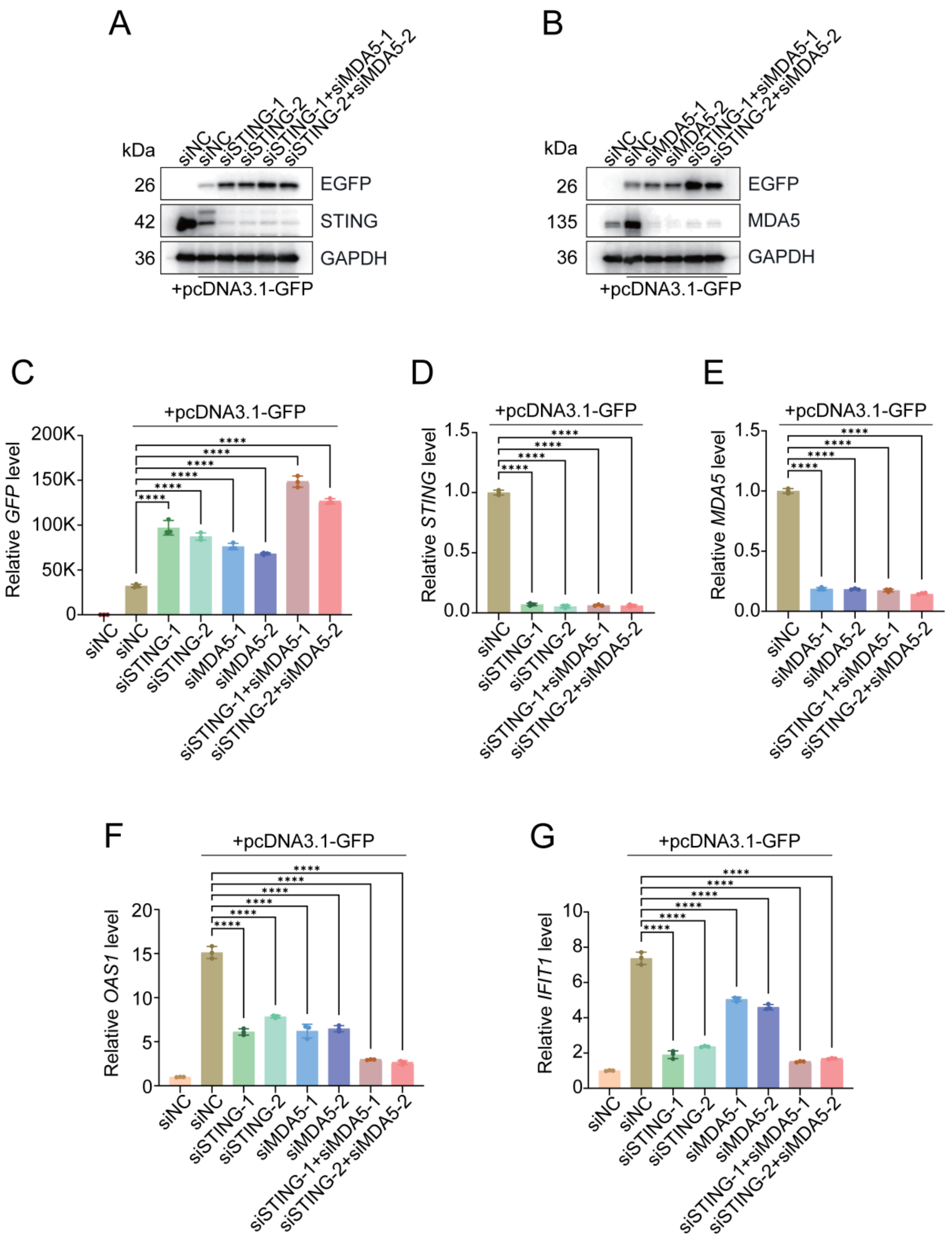

Figure S16. Elimination of the off-target effects of the siRNAs in the STING and MDA5 knockdown experiments.

Western blot analysis at 24 h showing the levels of STING **(A)** and MDA5 **(B)**, as well as the expression levels of EGFP, after STING or MDA5 were knocked down using two siRNAs with different targets in NCM460 cells transfected with pcDNA3.1-GFP plasmids. The siRNAs are designated -1 and -2, representing two different siRNA constructs. qPCR results at 24 h revealing the relative expression levels of GFP **(C)**, STING **(D)**, MDA5 **(E)**, OAS1 **(F)** and IFIT1 **(G)** compared with those of the GAPDH gene after knocking down STING or MDA5

using two siRNAs targeting different sites in NCM460 cells transfected with pcDNA3.1-GFP plasmids. Statistical P values were calculated using Dunnett's test after ANOVA, with siNC cells transfected with pcDNA3.1-GFP used as the control group. siNC: negative control siRNA. *$P < 0.05$, **$P < 0.01$, ***$P < 0.001$ and ****$P < 0.0001$. The siRNAs are designated -1 and -2, representing two different siRNA constructs. n=3 independent replicates.

A

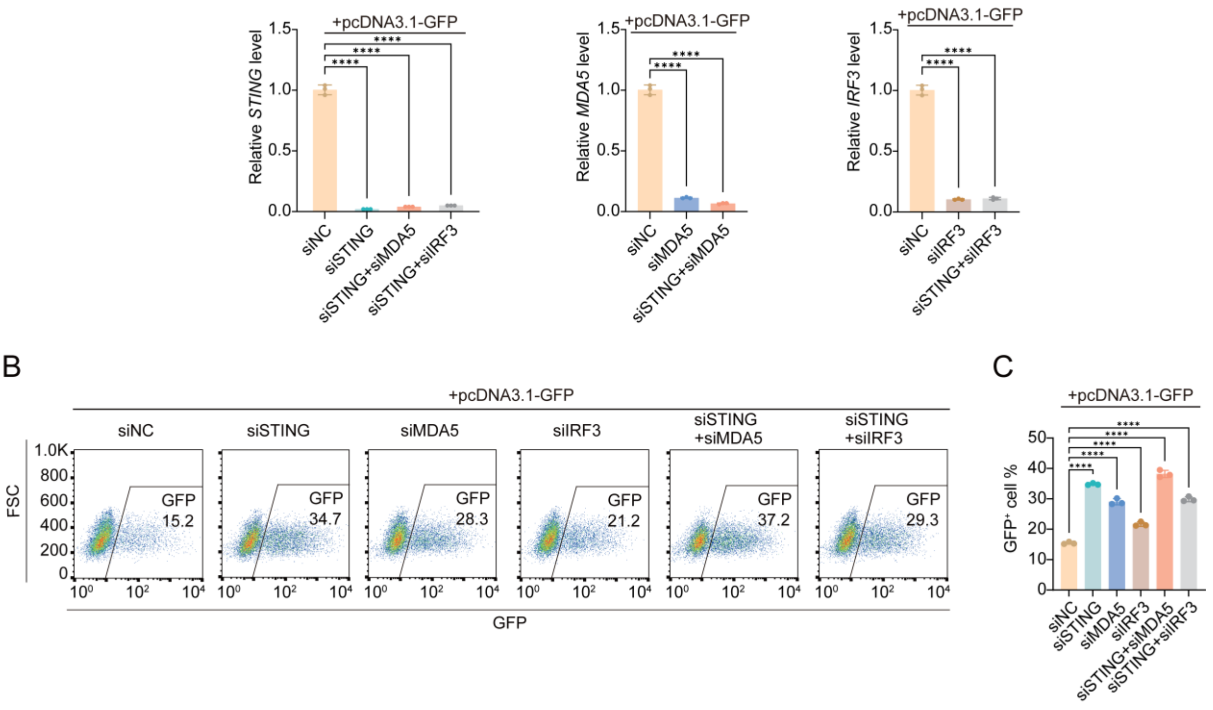

Figure S17. Transfection efficiency was improved with STING/MDA5 knockdown compared with STING/IRF3 knockdown.

(A) qPCR results at 24 h revealing the knockdown levels of siRNAs targeting STING, MDA5, and IRF3 in NCM460 cells transfected with pcDNA3.1-GFP plasmids. Statistical P values were calculated using Dunnett's test after ANOVA, with siNC cells transfected with pcDNA3.1-GFP used as the control group. siNC: negative control siRNA. *P < 0.05, **P < 0.01, ***P < 0.001 and ****P < 0.0001. n=3 independent replicates. **(B)** Flow cytometry revealing the impacts of cGAS, STING, MDA5, and IRF3 single and double (STING/MDA5 and STING/IRF3) knockdown in pcDNA3.1-GFP-transfected NCM460 cells. NC: negative control. FSC: forward scatter. n=3 independent replicates. **(C)** GFP^{+} cell ratio from the flow cytometry assay in (B). Statistical P values were calculated using Dunnett's test following ANOVA, with siNC cells transfected with pcDNA3.1-GFP used as the control group. siNC: negative control siRNA. *P < 0.05, **P < 0.01, ***P < 0.001 and ****P < 0.0001. n=3 independent replicates.

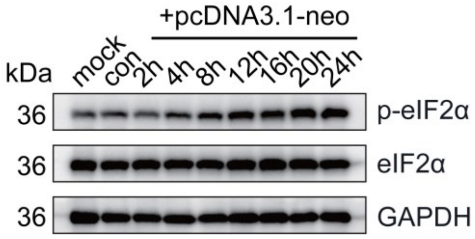

Figure S18. Western blots of eIF2α and p-eIF2α levels.

Western blot assay examining p-eIF2α, eIF2α, and GAPDH levels in HEK293T cells transfected with the pcDNA3.1-neo plasmid at various time points. con: control groups treated with only the transfection reagent. mock: wild-type cells.

Table S1. Common IIR genes identified in low-transfection-efficiency cells transfected with linear DNAs

Genes activated by linear pcDNA3.1-neo plasmids	Genes activated by linear pre-gRNA plasmids
EIF2AK2	EIF2AK2
OAS1	OAS1
RIGI	RIGI
OAS3	OAS3
MDA5	MDA5
STAT1	STAT1
IFIT3	IFIT3
IFIT2	IFIT2
IFI6	IFI6
BST2	BST2
SHFL	SHFL
RSAD2	RSAD2
OASL	OASL
DDX60	DDX60
IFI44L	IFI44
IFI44	PARP9
PARP9	HERC5
NLRC5	NLRC5
MX1	MX1
DTX3L	DTX3L
IFI27	IFI27
LGALS9	LGALS9
USP18	USP18
IRF7	IRF7
IFIT1	IFIT1
ISG15	ISG15
CCL5	

Table S2. Unique upregulated H3K27ac ChIP-seq peaks in HeLa cells transfected with foreign DNA

Upregulated peaks activated by pcDNA3.1-neo plasmids					
chr1:10659789-10660189	chr11:35289431-35289831	chr16:28222626-28223026	chr2:241377773-241378173	chr4:140273062-140273462	chr6:88415753-88416153
chr1:109262660-109263060	chr11:36531515-36531915	chr16:2845516-2845916	chr2:28022397-28022797	chr4:14291538-14291938	chr6:9789838-9790238
chr1:110461729-110462129	chr11:393859-394259	chr16:29973586-29973986	chr2:28519597-28519997	chr4:15010124-15010524	chr7:100781578-100781978
chr1:113277971-113278371	chr11:43977693-43978093	chr16:30671471-30671871	chr2:28631236-28631636	chr4:15044197-15044597	chr7:101426694-101427094
chr1:115297289-115297689	chr11:45920934-45921334	chr16:3071011-3071411	chr2:29120088-29120488	chr4:169237838-169238238	chr7:104738318-104738718
chr1:115709499-115709899	chr11:46270401-46270801	chr16:31075991-31076391	chr2:42569330-42569730	chr4:169242373-169242773	chr7:116512777-116513177
chr1:11881728-11882128	chr11:47198093-47198493	chr16:31150181-31150581	chr2:45581193-45581593	chr4:169355914-169356314	chr7:123438315-123438715
chr1:12050082-12050482	chr11:47573884-47574284	chr16:31276275-31276675	chr2:48560843-48561243	chr4:174267499-174267899	chr7:123486387-123486787
chr1:1240190-1240590	chr11:57078569-57078969	chr16:4425918-4426318	chr2:53314723-53315123	chr4:17579211-17579611	chr7:131236904-131237304
chr1:153538157-153538557	chr11:614409-614809	chr16:53795731-53796131	chr2:54830414-54830814	chr4:177196024-177196424	chr7:131750788-131751188
chr1:154405170-154405570	chr11:61657843-61658243	chr16:57480821-57481221	chr2:58347619-58348019	chr4:177466710-177467110	chr7:134232225-134232625
chr1:154580730-154581130	chr11:62355668-62356068	chr16:578467-578867	chr2:65058684-65059084	chr4:187467521-187467921	chr7:135194521-135194921
chr1:154941513-154941913	chr11:63612404-63612804	chr16:585323-585723	chr2:70203481-70203881	chr4:187468706-187469106	chr7:138758234-138758634
chr1:155953553-155953953	chr11:64102260-64102660	chr16:69498793-69499193	chr2:70295203-70295603	chr4:22740986-22741386	chr7:138779247-138779647
chr1:156866623-156867023	chr11:64691750-64692150	chr16:69499492-69499892	chr2:70298100-70298500	chr4:24583374-24583774	chr7:138786669-138787069
chr1:16011216-16011616	chr11:66247342-66247742	chr16:71842671-71843071	chr2:70338994-70339394	chr4:36843279-36843679	chr7:138797040-138797440
chr1:160175698-160176098	chr11:66406089-66406489	chr16:81861213-81861613	chr2:70363015-70363415	chr4:41205116-41205516	chr7:1506390-1506790
chr1:160231991-160232391	chr11:66696258-66696658	chr16:81862081-81862481	chr2:73461395-73461795	chr4:54872106-54872506	chr7:152131704-152132104
chr1:161195830-161196230	chr11:66845738-66846138	chr16:81879219-81879619	chr2:74730486-74730886	chr4:56169555-56169955	chr7:155458456-155458856
chr1:165853827-165854227	chr11:69070738-69071138	chr16:84763726-84764126	chr2:81885770-81886170	chr4:56600304-56600704	chr7:158699524-158699924
chr1:171436946-171437346	chr11:72960222-72960622	chr16:88850044-88850444	chr2:85518746-85519146	chr4:5760077-5760477	chr7:18203142-18203542
chr1:176002651-176003051	chr11:78236640-78237040	chr16:89645182-89645582	chr2:85581251-85581651	chr4:57848282-57848682	chr7:2003349-2003749
chr1:180742688-180743088	chr11:85370088-85370488	chr16:90061835-90062235	chr2:85725942-85726342	chr4:72978757-72979157	chr7:20415629-20416029
chr1:182838290-182838690	chr11:9601029-9601429	chr17:1302983-1303383	chr2:88899958-88900358	chr4:73943777-73944177	chr7:28149656-28150056
chr1:183430211-183430611	chr11:9922747-9923147	chr17:1493462-1493862	chr2:9569130-9569530	chr4:84308592-84308992	chr7:28150380-28150780
chr1:183430831-183431231	chr12:113375637-113376037	chr17:1552991-1553391	chr2:97006224-97006624	chr4:89378602-89379002	chr7:30208490-30208890
chr1:193291588-193291988	chr12:116819289-116819689	chr17:16769751-16770151	chr20:18507011-18507411	chr4:99850005-99850405	chr7:30590410-30590810
chr1:198227620-198228020	chr12:121581311-121581711	chr17:16946494-16946894	chr20:2451016-2451416	chr5:102091307-102091707	chr7:32546062-32546462
chr1:202168276-202168676	chr12:122230145-122230545	chr17:17009487-17009887	chr20:30743062-30743462	chr5:102097754-102098154	chr7:43599790-43600190
chr1:203632694-203633094	chr12:123314956-123315356	chr17:17013705-17014105	chr20:33509793-33510193	chr5:10388469-10388869	chr7:50869697-50870097
chr1:204251657-204252057	chr12:12966247-12966647	chr17:17722574-17722974	chr20:35439866-35440266	chr5:10577839-10578239	chr7:50880848-50881248
chr1:208352941-208353341	chr12:133022210-133022610	chr17:18089642-18090042	chr20:4116475-4116875	chr5:10624696-10625096	chr7:55172206-55172606
chr1:212782234-212782634	chr12:14366712-14367112	chr17:18164237-18164637	chr20:43961829-43962229	chr5:10760102-10760502	chr7:56029196-56029596
chr1:213241870-213242270	chr12:14455425-14455825	chr17:18906761-18907161	chr20:46250942-46251342	chr5:112209901-112210301	chr7:5741244-5741644
chr1:213246416-213246816	chr12:1579018-1579418	chr17:2497486-2497886	chr20:47892904-47893304	chr5:112211927-112212327	chr7:578203-578603
chr1:21411100-21411500	chr12:22004890-22005290	chr17:30483851-30484251	chr20:48304391-48304791	chr5:115174448-115174848	chr7:6469318-6469718

chr1:21847890-21848290	chr12:26208881-26209281	chr17:30518669-30519069	chr20:48323498-48323898	chr5:125695857-125696257	chr7:66056610-66057010
chr1:2186885-2187285	chr12:28932620-28933020	chr17:37170974-37171374	chr20:48325934-48326334	chr5:128113098-128113498	chr7:87483266-87483666
chr1:219971943-219972343	chr12:38782877-38783277	chr17:38332203-38332603	chr20:48434556-48434956	chr5:132298319-132298719	chr7:87513451-87513851
chr1:220292232-220292632	chr12:38786079-38786479	chr17:38801077-38801477	chr20:48687654-48688054	chr5:133303968-133304368	chr7:92746292-92746692
chr1:222828575-222828975	chr12:38941591-38941991	chr17:39845133-39845533	chr20:48758473-48758873	chr5:133339374-133339774	chr7:946489-946889
chr1:223914583-223914983	chr12:50930934-50931334	chr17:40439755-40440155	chr20:48809689-48810089	chr5:137335952-137336352	chr7:976315-976715
chr1:231346764-231347164	chr12:52595018-52595418	chr17:41156174-41156574	chr20:56885253-56885653	chr5:139678308-139678708	chr7:99158048-99158448
chr1:2343368-2343768	chr12:60366968-60367368	chr17:41602600-41603000	chr20:57267239-57267639	chr5:141037332-141037732	chr8:100960738-100961138
chr1:23435729-23436129	chr12:6276748-6277148	chr17:47592621-47593021	chr20:60925292-60925692	chr5:141488799-141489199	chr8:107405237-107405637
chr1:23436464-23436864	chr12:64855745-64856145	chr17:48719633-48720033	chr20:61505377-61505777	chr5:142329401-142329801	chr8:119634112-119634512
chr1:235575658-235576058	chr12:6495991-6496391	chr17:48796886-48797286	chr20:62317953-62318353	chr5:147763668-147764068	chr8:128315771-128316171
chr1:236694545-236694945	chr12:65073710-65074110	chr17:5326187-5326587	chr21:16406518-16406918	chr5:149127897-149128297	chr8:128822130-128822530
chr1:237922303-237922703	chr12:905069-905469	chr17:59582343-59582743	chr21:30047324-30047724	chr5:150431613-150432013	chr8:128829165-128829565
chr1:240568717-240569117	chr12:91800947-91801347	chr17:65505880-65506280	chr21:35104457-35104857	chr5:150455509-150455909	chr8:128899662-128900062
chr1:245878948-245879348	chr12:95594625-95595025	chr17:7153486-7153886	chr21:42796516-42796916	chr5:150494601-150495001	chr8:128956860-128957260
chr1:246792484-246792884	chr12:97113868-97114268	chr17:72199944-72200344	chr21:45153544-45153944	chr5:154135897-154136297	chr8:134534478-134534878
chr1:24708094-24708494	chr13:100034891-100035291	chr17:72987915-72988315	chr21:45187944-45188344	chr5:158397539-158397939	chr8:135814447-135814847
chr1:24735601-24736001	chr13:104967444-104967844	chr17:73718026-73718426	chr21:47563601-47564001	chr5:159354880-159355280	chr8:141844534-141844934
chr1:26607323-26607723	chr13:110422310-110422710	chr17:76528760-76529160	chr22:22221001-22221401	chr5:163655647-163656047	chr8:142145866-142146266
chr1:27175425-27175825	chr13:111609474-111609874	chr17:8286189-8286589	chr22:25516175-25516575	chr5:163763161-163763561	chr8:142147472-142147872
chr1:27179911-27180311	chr13:114540323-114540723	chr18:18874995-18875395	chr22:28281938-28282338	chr5:166634441-166634841	chr8:142174749-142175149
chr1:27986743-27987143	chr13:114745118-114745518	chr18:23889839-23890239	chr22:33091898-33092298	chr5:166636238-166636638	chr8:144105137-144105537
chr1:28298056-28298456	chr13:114780651-114781051	chr18:2592989-2593389	chr22:38703794-38704194	chr5:17228086-17228486	chr8:144106019-144106419
chr1:28899068-28899468	chr13:33112584-33112984	chr18:46065519-46065919	chr22:39092847-39093247	chr5:17231803-17232203	chr8:144120358-144120758
chr1:31538155-31538555	chr13:41234048-41234448	chr18:47804553-47804953	chr22:41748299-41748699	chr5:17238906-17239306	chr8:144676311-144676711
chr1:32400488-32400888	chr13:46066420-46066820	chr18:48500020-48500420	chr22:42770708-42771108	chr5:173282011-173282411	chr8:145681666-145682066
chr1:32489198-32489598	chr13:47177692-47178092	chr18:57549370-57549770	chr22:43551816-43552216	chr5:175840426-175840826	chr8:145981121-145981521
chr1:32566938-32567338	chr13:49619099-49619499	chr18:57552700-57553100	chr22:43555476-43555876	chr5:175875321-175875721	chr8:19472408-19472808
chr1:32859619-32860019	chr13:49627117-49627517	chr18:57567536-57567936	chr22:46299199-46299599	chr5:175892980-175893380	chr8:22025794-22026194
chr1:33225736-33226136	chr13:49720118-49720518	chr18:57572004-57572404	chr22:46773713-46774113	chr5:21679202-21679602	chr8:22526123-22526523
chr1:33476392-33476792	chr13:53712977-53713377	chr18:58644495-58644895	chr22:47170039-47170439	chr5:25558724-25559124	chr8:23018742-23019142
chr1:36621125-36621525	chr13:60737580-60737980	chr18:72458388-72458788	chr22:50328478-50328878	chr5:28301818-28302218	chr8:23420335-23420735
chr1:37917706-37918106	chr13:73634507-73634907	chr18:76916367-76916767	chr22:50342481-50342881	chr5:34923787-34924187	chr8:26014793-26015193
chr1:37999768-38000168	chr13:93243137-93243537	chr18:938685-939085	chr3:10065514-10065914	chr5:37197468-37197868	chr8:26184685-26185085
chr1:39456423-39456823	chr14:102552539-102552939	chr19:1017652-1018052	chr3:103998266-103998666	chr5:38851871-38852271	chr8:38771752-38772152
chr1:41468947-41469347	chr14:103574879-103575279	chr19:1185545-1185945	chr3:105087023-105087423	chr5:38853771-38854171	chr8:41008806-41009206
chr1:59897477-59897877	chr14:103726721-103727121	chr19:12401566-12401966	chr3:11272135-11272535	chr5:38875511-38875911	chr8:54819684-54820084
chr1:65011556-65011956	chr14:105156614-105157014	chr19:13123843-13124243	chr3:11285466-11285866	chr5:40405643-40406043	chr8:580953-581353
chr1:65361306-65361706	chr14:21732008-21732408	chr19:18145834-18146234	chr3:117341096-117341496	chr5:40408472-40408872	chr8:61621520-61621920

chr1:65416529-65416929	chr14:23562447-23562847	chr19:18631718-18632118	chr3:119854630-119855030	chr5:40666949-40667349	chr9:100466149-100466549
chr1:65501871-65502271	chr14:23776001-23776401	chr19:19515800-19516200	chr3:121201755-121202155	chr5:40667582-40667982	chr9:103191177-103191577
chr1:68036469-68036869	chr14:24721379-24721779	chr19:2087219-2087619	chr3:122400301-122400701	chr5:52333000-52333400	chr9:107815054-107815454
chr1:89321313-89321713	chr14:38058579-38058979	chr19:2114202-2114602	chr3:124129015-124129415	chr5:55624711-55625111	chr9:111774348-111774748
chr1:90312121-90312521	chr14:53372804-53373204	chr19:2168547-2168947	chr3:128445003-128445403	chr5:57012872-57013272	chr9:115915741-115916141
chr1:92206084-92206484	chr14:55240342-55240742	chr19:3606897-3607297	chr3:129147565-129147965	chr5:67610306-67610706	chr9:119041893-119042293
chr1:93297536-93297936	chr14:62197805-62198205	chr19:4058574-4058974	chr3:129513456-129513856	chr5:67853269-67853669	chr9:124461941-124462341
chr1:94099779-94100179	chr14:62222313-62222713	chr19:41082677-41083077	chr3:132151124-132151524	chr5:71659172-71659572	chr9:125540301-125540701
chr1:94128360-94128760	chr14:65174741-65175141	chr19:43265146-43265546	chr3:132205289-132205689	chr5:74058877-74059277	chr9:127549985-127550385
chr1:951587-951987	chr14:69446205-69446605	chr19:43769359-43769759	chr3:132209220-132209620	chr5:78857270-78857670	chr9:129961351-129961751
chr10:100220783-100221183	chr14:71132396-71132796	chr19:44173200-44173600	chr3:134202170-134202570	chr5:7903956-7904356	chr9:130299949-130300349
chr10:101753973-101754373	chr14:71791197-71791597	chr19:44808721-44809121	chr3:136069237-136069637	chr5:93959410-93959810	chr9:130565855-130566255
chr10:104370853-104371253	chr14:75130068-75130468	chr19:45198403-45198803	chr3:137762459-137762859	chr5:95296408-95296808	chr9:130698029-130698429
chr10:114010667-114011067	chr14:77769727-77770127	chr19:4694707-4695107	chr3:138413029-138413429	chr6:10760524-10760924	chr9:131185300-131185700
chr10:120850763-120851163	chr14:93252276-93252676	chr19:48281616-48282016	chr3:146174514-146174914	chr6:111808385-111808785	chr9:131397782-131398182
chr10:120891169-120891569	chr15:100244213-100244613	chr19:49466789-49467189	chr3:148964333-148964733	chr6:111809332-111809732	chr9:132474644-132475044
chr10:21476478-21476878	chr15:31781398-31781798	chr19:53225373-53225773	chr3:149363594-149363994	chr6:114292222-114292622	chr9:132504167-132504567
chr10:22566897-22567297	chr15:34446778-34447178	chr19:752134-752534	chr3:15205309-15205709	chr6:138197296-138197696	chr9:133454535-133454935
chr10:29453101-29453501	chr15:36924049-36924449	chr19:9129048-9129448	chr3:159656183-159656583	chr6:146865814-146866214	chr9:137555587-137555987
chr10:309966-310366	chr15:40482391-40482791	chr19:9649247-9649647	chr3:169697617-169698017	chr6:149884716-149885116	chr9:139801143-139801543
chr10:3826075-3826475	chr15:41186803-41187203	chr2:102383361-102383761	chr3:171811368-171811768	chr6:15285400-15285800	chr9:17613442-17613842
chr10:3869661-3870061	chr15:45353171-45353571	chr2:110348567-110348967	chr3:184017250-184017650	chr6:159193578-159193978	chr9:17894441-17894841
chr10:3871569-3871969	chr15:49428472-49428872	chr2:113380833-113381233	chr3:186501012-186501412	chr6:166383075-166383475	chr9:21814199-21814599
chr10:71921080-71921480	chr15:49447430-49447830	chr2:12951608-12952008	chr3:187906893-187907293	chr6:24721087-24721487	chr9:21815531-21815931
chr10:71927679-71928079	chr15:55920242-55920642	chr2:161763780-161764180	chr3:195597162-195597562	chr6:26388475-26388875	chr9:21990555-21990955
chr10:72027521-72027921	chr15:56329867-56330267	chr2:165970997-165971397	chr3:19987886-19988286	chr6:33636396-33636796	chr9:32165974-32166374
chr10:73823716-73824116	chr15:60761226-60761626	chr2:174837683-174838083	chr3:33414132-33414532	chr6:34576770-34577170	chr9:32166611-32167011
chr10:74371950-74372350	chr15:66748730-66749130	chr2:191876582-191876982	chr3:44510288-44510688	chr6:34664449-34664849	chr9:32529249-32529649
chr10:76975470-76975870	chr15:66914154-66914554	chr2:192439364-192439764	chr3:47767363-47767763	chr6:36571715-36572115	chr9:32530004-32530404
chr10:81026172-81026572	chr15:68405664-68406064	chr2:197013627-197014027	chr3:49130425-49130825	chr6:36720513-36720913	chr9:33022542-33022942
chr10:88730758-88731158	chr15:74280078-74280478	chr2:201248087-201248487	chr3:49675568-49675968	chr6:41748651-41749051	chr9:33123550-33123950
chr10:92980651-92981051	chr15:75248210-75248610	chr2:203879004-203879404	chr3:49676156-49676556	chr6:42110462-42110862	chr9:34231894-34232294
chr10:97049664-97050064	chr15:75423082-75423482	chr2:207077234-207077634	chr3:49938238-49938638	chr6:43445543-43445943	chr9:36584912-36585312
chr10:99302187-99302587	chr15:79088248-79088648	chr2:208675169-208675569	chr3:5166875-5167275	chr6:43603579-43603979	chr9:37953835-37954235
chr10:99440215-99440615	chr15:81069109-81069509	chr2:213990645-213991045	chr3:52029563-52029963	chr6:43655034-43655434	chr9:73515529-73515929
chr11:100579799-100580199	chr15:82438975-82439375	chr2:216844754-216845154	chr3:52725993-52726393	chr6:43766163-43766563	chr9:85642124-85642524
chr11:102171703-102172103	chr15:89171373-89171773	chr2:219446973-219447373	chr3:57790631-57791031	chr6:44308030-44308430	chr9:94445287-94445687
chr11:110024836-110025236	chr15:90754751-90755151	chr2:220110166-220110566	chr3:64123409-64123809	chr6:48000275-48000675	chr9:95871715-95872115
chr11:11863924-11864324	chr15:93464751-93465151	chr2:220138967-220139367	chr3:8019273-8019673	chr6:4934287-4934687	chr9:96232721-96233121

chr11:118798787-118799187	chr16:121792-122192	chr2:223288002-223288402	chr3:9437692-9438092	chr6:5125491-5125891	chr9:96328977-96329377
chr11:126216341-126216741	chr16:18801325-18801725	chr2:230687200-230687600	chr3:99282291-99282691	chr6:52455121-52455521	chrX:102911804-102912204
chr11:17192677-17193077	chr16:20890243-20890643	chr2:232650740-232651140	chr4:10115696-10116096	chr6:53179874-53180274	chrX:128800419-128800819
chr11:17484796-17485196	chr16:20908326-20908726	chr2:235001050-235001450	chr4:110092639-110093039	chr6:54667215-54667615	chrX:43549354-43549754
chr11:19965547-19965947	chr16:21314101-21314501	chr2:236635616-236636016	chr4:111208505-111208905	chr6:56728504-56728904	chrX:44927495-44927895
chr11:19967023-19967423	chr16:23156905-23157305	chr2:23704012-23704412	chr4:123799734-123800134	chr6:64350093-64350493	chrX:44928099-44928499
chr11:210113-210513	chr16:2564715-2565115	chr2:238907048-238907448	chr4:129750000-129750400	chr6:71972680-71973080	chrX:7289206-7289606
chr11:313443-313843	chr16:28193013-28193413	chr2:241313305-241313705	chr4:134834176-134834576	chr6:82691427-82691827	chrX:9833726-9834126

Note: The notation "chromosome:start_position-end_position" signifies the peak location.

Upregulated peaks activated by linear pcDNA3.1-neo plasmids					
chr1:100069651-100070051	chr11:17281232-17281632	chr15:75953673-75954073	chr2:203000000-203000400	chr3:88080764-88081164	chr6:53330321-53330721
chr1:101313214-101313614	chr11:19298361-19298761	chr15:84416216-84416616	chr2:20638659-20639059	chr3:88191157-88191557	chr6:56573020-56573420
chr1:102410469-102410869	chr11:19975163-19975563	chr15:89164875-89165275	chr2:20739461-20739861	chr3:93687944-93688344	chr6:6803533-6803933
chr1:10588082-10588482	chr11:20385598-20385998	chr15:89173791-89174191	chr2:208393886-208394286	chr3:93723298-93723698	chr6:68071136-68071536
chr1:109246859-109247259	chr11:20395907-20396307	chr15:89672599-89672999	chr2:20850412-20850812	chr3:9773098-9773498	chr6:73234022-73234422
chr1:11072376-11072776	chr11:2951088-2951488	chr15:89892240-89892640	chr2:208541560-208541960	chr3:98690344-98690744	chr6:73270484-73270884
chr1:111505994-111506394	chr11:30344643-30345043	chr15:89945340-89945740	chr2:208675768-208676168	chr3:98705283-98705683	chr6:73289798-73290198
chr1:112298476-112298876	chr11:3043922-3044322	chr15:90085284-90085684	chr2:210360069-210360469	chr3:99264632-99265032	chr6:7542604-7543004
chr1:113162397-113162797	chr11:3122226-3122626	chr15:90436754-90437154	chr2:214990321-214990721	chr4:104013590-104013990	chr6:75992427-75992827
chr1:113408466-113408866	chr11:314127-314527	chr15:90548190-90548590	chr2:216176280-216176680	chr4:110092062-110092462	chr6:82463098-82463498
chr1:115000565-115000965	chr11:31634350-31634750	chr15:90905619-90906019	chr2:218560082-218560482	chr4:111790710-111791110	chr6:86071373-86071773
chr1:115711715-115712115	chr11:32159287-32159687	chr15:92396077-92396477	chr2:219135329-219135729	chr4:123668059-123668459	chr6:86113639-86114039
chr1:116088086-116088486	chr11:32915962-32916362	chr15:92496609-92497009	chr2:219652748-219653148	chr4:123707753-123708153	chr6:86159922-86160322
chr1:11689555-11689955	chr11:33060775-33061175	chr15:93348115-93348515	chr2:220252104-220252504	chr4:123714848-123715248	chr6:90927716-90928116
chr1:116919845-116920245	chr11:34255910-34256310	chr16:11645726-11646126	chr2:224814576-224814976	chr4:126119821-126120221	chr6:93688408-93688808
chr1:117603004-117603404	chr11:3443157-3443557	chr16:11928543-11928943	chr2:230272517-230272917	chr4:129314407-129314807	chr7:100101321-100101721
chr1:11850604-11851004	chr11:35684507-35684907	chr16:1346701-1347101	chr2:230785729-230786129	chr4:129748226-129748626	chr7:100486985-100487385
chr1:119358066-119358466	chr11:36276564-36276964	chr16:1347237-1347637	chr2:231084386-231084786	chr4:131484709-131485109	chr7:100728908-100729308
chr1:119530062-119530462	chr11:36383178-36383578	chr16:15737090-15737490	chr2:231422288-231422688	chr4:133148750-133149150	chr7:101425937-101426337
chr1:1208503-1208903	chr11:43942601-43943001	chr16:16070555-16070955	chr2:232026390-232026790	chr4:152024369-152024769	chr7:101525682-101526082
chr1:1279759-1280159	chr11:44052417-44052817	chr16:1795908-1796308	chr2:232507821-232508221	chr4:154354437-154354837	chr7:101707415-101707815
chr1:150737722-150738122	chr11:45576391-45576791	chr16:18938141-18938541	chr2:232514884-232515284	chr4:159456683-159457083	chr7:10214527-10214927
chr1:151167229-151167629	chr11:460240-460640	chr16:18957459-18957859	chr2:232515598-232515998	chr4:162749078-162749478	chr7:102937823-102938223
chr1:151583925-151584325	chr11:46402338-46402738	chr16:20421786-20422186	chr2:237657373-237657773	chr4:168824710-168825110	chr7:104653272-104653672
chr1:151956202-151956602	chr11:46582581-46582981	chr16:2059944-2060344	chr2:237864659-237865059	chr4:169542620-169543020	chr7:104859581-104859981
chr1:152020695-152021095	chr11:47291190-47291590	chr16:23157394-23157794	chr2:238601373-238601773	chr4:172124842-172125242	chr7:105513335-105513735

chr1:15272450-15272850	chr11:47358559-47358959	chr16:24537081-24537481	chr2:240323023-240323423	chr4:175075372-175075772	chr7:114459421-114459821
chr1:153545369-153545769	chr11:47429272-47429672	chr16:24550839-24551239	chr2:241195234-241195634	chr4:177159598-177159998	chr7:116355685-116356085
chr1:154192735-154193135	chr11:47534404-47534804	chr16:2802155-2802555	chr2:241324394-241324794	chr4:183413293-183413693	chr7:122559531-122559931
chr1:154578064-154578464	chr11:48129041-48129441	chr16:2827464-2827864	chr2:26256930-26257330	chr4:186629517-186629917	chr7:123410801-123411201
chr1:154580200-154580600	chr11:5705922-5706322	chr16:2844753-2845153	chr2:27654471-27654871	chr4:186990135-186990535	chr7:126214593-126214993
chr1:154909420-154909820	chr11:5707113-5707513	chr16:28503334-28503734	chr2:28631706-28632106	chr4:187656698-187657098	chr7:128249179-128249579
chr1:154946370-154946770	chr11:57333387-57333787	chr16:30089360-30089760	chr2:31556858-31557258	chr4:22669435-22669835	chr7:128503017-128503417
chr1:155107774-155108174	chr11:57334627-57335027	chr16:30389195-30389595	chr2:3583786-3584186	chr4:22670306-22670706	chr7:131318439-131318839
chr1:155293663-155294063	chr11:58538608-58539008	chr16:3070527-3070927	chr2:37153298-37153698	chr4:2537268-2537668	chr7:135259844-135260244
chr1:155532544-155532944	chr11:58910231-58910631	chr16:31007977-31008377	chr2:39719016-39719416	chr4:26199170-26199570	chr7:135430260-135430660
chr1:155714986-155715386	chr11:59324134-59324534	chr16:31009181-31009581	chr2:42161195-42161595	chr4:2936557-2936957	chr7:137193217-137193617
chr1:156252262-156252662	chr11:60719793-60720193	chr16:31276994-31277394	chr2:45582166-45582566	chr4:3288017-3288417	chr7:138728708-138729108
chr1:156426529-156426929	chr11:61559760-61560160	chr16:31464474-31464874	chr2:46257116-46257516	chr4:36005878-36006278	chr7:138781176-138781576
chr1:158985517-158985917	chr11:61732070-61732470	chr16:31470731-31471131	chr2:47651535-47651935	chr4:38076355-38076755	chr7:138794090-138794490
chr1:159046732-159047132	chr11:61811694-61812094	chr16:3989438-3989838	chr2:48339121-48339521	chr4:38162268-38162668	chr7:138802526-138802926
chr1:159083714-159084114	chr11:63333099-63333499	chr16:4186489-4186889	chr2:48347676-48348076	chr4:39355289-39355689	chr7:138803375-138803775
chr1:160489894-160490294	chr11:64013461-64013861	chr16:46863805-46864205	chr2:54747919-54748319	chr4:3956507-3956907	chr7:139168538-139168938
chr1:16125183-16125583	chr11:64052269-64052669	chr16:47007396-47007796	chr2:58340374-58340774	chr4:41645136-41645536	chr7:139761986-139762386
chr1:161356056-161356456	chr11:64684212-64684612	chr16:48657435-48657835	chr2:65034569-65034969	chr4:48727946-48728346	chr7:139762776-139763176
chr1:16175518-16175918	chr11:65430716-65431116	chr16:50733341-50733741	chr2:69983468-69983868	chr4:54871670-54872070	chr7:150010150-150010550
chr1:162350889-162351289	chr11:65585107-65585507	chr16:56573648-56574048	chr2:70025635-70026035	chr4:56050521-56050921	chr7:150755268-150755668
chr1:162508900-162509300	chr11:65695200-65695600	chr16:56776238-56776638	chr2:7004842-7005242	chr4:56731475-56731875	chr7:151039161-151039561
chr1:16275819-16276219	chr11:66094634-66095034	chr16:57337793-57338193	chr2:7006092-7006492	chr4:5787493-5787893	chr7:151216398-151216798
chr1:167635266-167635666	chr11:67113695-67114095	chr16:57349239-57349639	chr2:7017298-7017698	chr4:62889062-62889462	chr7:151541228-151541628
chr1:169863060-169863460	chr11:67807754-67808154	chr16:58718291-58718691	chr2:70212654-70213054	chr4:64803666-64804066	chr7:151722322-151722722
chr1:170137290-170137690	chr11:69045924-69046324	chr16:61923974-61924374	chr2:70296582-70296982	chr4:74088317-74088717	chr7:154719717-154720117
chr1:171410668-171411068	chr11:69048181-69048581	chr16:66930775-66931175	chr2:71612428-71612828	chr4:76949534-76949934	chr7:155457979-155458379
chr1:171437729-171438129	chr11:69069726-69070126	chr16:67555164-67555564	chr2:74056316-74056716	chr4:77134316-77134716	chr7:157130194-157130594
chr1:171454620-171455020	chr11:69457706-69458106	chr16:67867192-67867592	chr2:74405646-74406046	chr4:77135132-77135532	chr7:1572126-1572526
chr1:173501514-173501914	chr11:70049949-70050349	chr16:67892124-67892524	chr2:74794576-74794976	chr4:7966737-7967137	chr7:158657069-158657469
chr1:173640293-173640693	chr11:71498813-71499213	chr16:68298861-68299261	chr2:75071484-75071884	chr4:81101470-81101870	chr7:1607216-1607616
chr1:178096273-178096673	chr11:73490214-73490614	chr16:68789688-68790088	chr2:85280607-85281007	chr4:8185006-8185406	chr7:17182239-17182639
chr1:178113227-178113627	chr11:75479421-75479821	chr16:69564059-69564459	chr2:85737820-85738220	chr4:83787172-83787572	chr7:18548810-18549210
chr1:17895931-17896331	chr11:75945747-75946147	chr16:70838316-70838716	chr2:86121100-86121500	chr4:89303730-89304130	chr7:2001729-2002129
chr1:179096502-179096902	chr11:76401685-76402085	chr16:72223780-72224180	chr2:86579433-86579833	chr5:10018368-10018768	chr7:20413616-20414016
chr1:180329132-180329532	chr11:76481393-76481793	chr16:73102662-73103062	chr2:86790038-86790438	chr5:100225729-100226129	chr7:20423928-20424328
chr1:180540850-180541250	chr11:79874203-79874603	chr16:74775124-74775524	chr2:88913350-88913750	chr5:10249955-10250355	chr7:20497719-20498119
chr1:181059191-181059591	chr11:79879028-79879428	chr16:81040449-81040849	chr2:97151568-97151968	chr5:104194826-104195226	chr7:2058838-2059238
chr1:183559150-183559550	chr11:80126254-80126654	chr16:85028133-85028533	chr2:97282691-97283091	chr5:10563778-10564178	chr7:22617434-22617834

chr1:186719697-186720097	chr11:85827451-85827851	chr16:85063374-85063774	chr20:11524294-11524694	chr5:10595594-10595994	chr7:23450443-23450843
chr1:186788493-186788893	chr11:86178212-86178612	chr16:88276073-88276473	chr20:1306026-1306426	chr5:107338655-107339055	chr7:2393802-2394202
chr1:186943634-186944034	chr11:86803480-86803880	chr16:89461130-89461530	chr20:1459512-1459912	chr5:112383285-112383685	chr7:24781198-24781598
chr1:186944323-186944723	chr11:8980766-8981166	chr16:90061407-90061807	chr20:17661262-17661662	chr5:114786298-114786698	chr7:25010578-25010978
chr1:19407450-19407850	chr11:9285898-9286298	chr17:11927979-11928379	chr20:18504959-18505359	chr5:114796608-114797008	chr7:25165237-25165637
chr1:196496877-196497277	chr11:95523578-95523978	chr17:16281497-16281897	chr20:1924060-1924460	chr5:114886683-114887083	chr7:25889073-25889473
chr1:196527784-196528184	chr12:104404686-104405086	chr17:17380421-17380821	chr20:23603889-23604289	chr5:114887295-114887695	chr7:26445385-26445785
chr1:198203736-198204136	chr12:104457561-104457961	chr17:17743511-17743911	chr20:23610024-23610424	chr5:114894700-114895100	chr7:28170231-28170631
chr1:201279750-201280150	chr12:104680580-104680980	chr17:20944377-20944777	chr20:25290376-25290776	chr5:115276599-115276999	chr7:28195801-28196201
chr1:201462367-201462767	chr12:105065201-105065601	chr17:2362641-2363041	chr20:30148288-30148688	chr5:115367388-115367788	chr7:30209102-30209502
chr1:203527065-203527465	chr12:10517684-10518084	chr17:26592730-26593130	chr20:30300037-30300437	chr5:118696283-118696683	chr7:31787434-31787834
chr1:204288998-204289398	chr12:106620974-106621374	chr17:27482842-27483242	chr20:30495998-30496398	chr5:119741262-119741662	chr7:33066714-33067114
chr1:204380560-204380960	chr12:108152964-108153364	chr17:27972131-27972531	chr20:30697246-30697646	chr5:119793733-119794133	chr7:33814285-33814685
chr1:204417995-204418395	chr12:110013712-110014112	chr17:28008864-28009264	chr20:31333773-31334173	chr5:119959996-119960396	chr7:33861182-33861582
chr1:205091075-205091475	chr12:110450253-110450653	chr17:28443949-28444349	chr20:31408139-31408539	chr5:123652002-123652402	chr7:33864305-33864705
chr1:205680487-205680887	chr12:110939368-110939768	chr17:29379206-29379606	chr20:32235494-32235894	chr5:125778761-125779161	chr7:34694721-34695121
chr1:207058275-207058675	chr12:112568258-112568658	chr17:32510482-32510882	chr20:32321262-32321662	chr5:125782848-125783248	chr7:34695886-34696286
chr1:20834263-20834663	chr12:113344157-113344557	chr17:32515799-32516199	chr20:33680774-33681174	chr5:125807500-125807900	chr7:35500929-35501329
chr1:208357174-208357574	chr12:113376201-113376601	chr17:32641034-32641434	chr20:33975761-33976161	chr5:125818343-125818743	chr7:35503357-35503757
chr1:209161932-209162332	chr12:117294270-117294670	chr17:35208221-35208621	chr20:34287366-34287766	chr5:129877297-129877697	chr7:36684788-36685188
chr1:210046542-210046942	chr12:118498326-118498726	chr17:37886455-37886855	chr20:35575187-35575587	chr5:131132356-131132756	chr7:37768145-37768545
chr1:210057803-210058203	chr12:120639381-120639781	chr17:38883874-38884274	chr20:35579779-35580179	chr5:131700400-131700800	chr7:38502627-38503027
chr1:211765236-211765636	chr12:121122512-121122912	chr17:40481199-40481599	chr20:35918089-35918489	chr5:131833637-131834037	chr7:39988619-39989019
chr1:212731910-212732310	chr12:121464806-121465206	chr17:41437660-41438060	chr20:36671924-36672324	chr5:133706794-133707194	chr7:41150149-41150549
chr1:212769877-212770277	chr12:122230824-122231224	chr17:41546171-41546571	chr20:36899843-36900243	chr5:1342801-1343201	chr7:42109332-42109732
chr1:212925897-212926297	chr12:122803540-122803940	chr17:43050025-43050425	chr20:37418462-37418862	chr5:137879120-137879520	chr7:44836619-44837019
chr1:21476850-21477250	chr12:122948494-122948894	chr17:43317001-43317401	chr20:388893-389293	chr5:137911056-137911456	chr7:45019574-45019974
chr1:215292196-215292596	chr12:123163911-123164311	chr17:4752160-4752560	chr20:39676670-39677070	chr5:138609886-138610286	chr7:45771780-45772180
chr1:221623292-221623692	chr12:123315712-123316112	chr17:47723781-47724181	chr20:39706282-39706682	chr5:14109241-14109641	chr7:47589900-47590300
chr1:222161408-222161808	chr12:123431261-123431661	chr17:48474208-48474608	chr20:42086508-42086908	chr5:14164381-14164781	chr7:47708855-47709255
chr1:223071103-223071503	chr12:123722257-123722657	chr17:49337426-49337826	chr20:43963159-43963559	chr5:142389114-142389514	chr7:51331522-51331922
chr1:223403732-223404132	chr12:12419905-12420305	chr17:53654016-53654416	chr20:44137466-44137866	chr5:147566162-147566562	chr7:54899138-54899538
chr1:224075473-224075873	chr12:125400539-125400939	chr17:53804373-53804773	chr20:45946523-45946923	chr5:147722890-147723290	chr7:54972241-54972641
chr1:225610437-225610837	chr12:125423259-125423659	chr17:56061157-56061557	chr20:46116313-46116713	chr5:148003639-148004039	chr7:55035390-55035790
chr1:227751152-227751552	chr12:12714261-12714661	chr17:56063787-56064187	chr20:48146220-48146620	chr5:148293003-148293403	chr7:55088046-55088446
chr1:228354007-228354407	chr12:13079968-13080368	chr17:57152237-57152637	chr20:48227819-48228219	chr5:148381997-148382397	chr7:55133124-55133524
chr1:228674706-228675106	chr12:133405393-133405793	chr17:57696628-57697028	chr20:48300351-48300751	chr5:148452561-148452961	chr7:55142007-55142407
chr1:229644110-229644510	chr12:14369032-14369432	chr17:60630684-60631084	chr20:48404263-48404663	chr5:149119698-149120098	chr7:55433648-55434048
chr1:232841366-232841766	chr12:14460141-14460541	chr17:62223500-62223900	chr20:48422848-48423248	chr5:150460729-150461129	chr7:5595920-5596320

chr1:233656502-233656902	chr12:16034943-16035343	chr17:6659465-6659865	chr20:48753971-48754371	chr5:150467037-150467437	chr7:56031773-56032173
chr1:233791294-233791694	chr12:16051910-16052310	chr17:67246673-67247073	chr20:48843644-48844044	chr5:1516702-1517102	chr7:56032656-56033056
chr1:233804702-233805102	chr12:16541939-16542339	chr17:67264555-67264955	chr20:5058916-5059316	chr5:152737884-152738284	chr7:5862260-5862660
chr1:233818551-233818951	chr12:20757844-20758244	chr17:69981724-69982124	chr20:52390864-52391264	chr5:153344362-153344762	chr7:6145325-6145725
chr1:235696073-235696473	chr12:26102457-26102857	chr17:70706552-70706952	chr20:52446045-52446445	chr5:154158475-154158875	chr7:66205127-66205527
chr1:236958607-236959007	chr12:27167441-27167841	chr17:71287929-71288329	chr20:52824339-52824739	chr5:154209902-154210302	chr7:68445240-68445640
chr1:238225284-238225684	chr12:27484530-27484930	chr17:7211675-7212075	chr20:55043672-55044072	chr5:158177128-158177528	chr7:75987917-75988317
chr1:240448564-240448964	chr12:27536710-27537110	chr17:72209184-72209584	chr20:56882289-56882689	chr5:158302492-158302892	chr7:76583942-76584342
chr1:24126232-24126632	chr12:29769766-29770166	chr17:72987253-72987653	chr20:57657502-57657902	chr5:163429398-163429798	chr7:7805241-7805641
chr1:244462897-244463297	chr12:31805727-31806127	chr17:73084018-73084418	chr20:58495735-58496135	chr5:16656973-16657373	chr7:79847642-79848042
chr1:244465479-244465879	chr12:31900904-31901304	chr17:7339695-7340095	chr20:60696489-60696889	chr5:166707-167107	chr7:8029380-8029780
chr1:244763394-244763794	chr12:38785124-38785524	chr17:7359182-7359582	chr20:62203253-62203653	chr5:16936494-16936894	chr7:80928170-80928570
chr1:245023523-245023923	chr12:44240958-44241358	chr17:73606600-73607000	chr20:62496833-62497233	chr5:169398355-169398755	chr7:81833762-81834162
chr1:246117202-246117602	chr12:45594150-45594550	chr17:73781235-73781635	chr20:62504360-62504760	chr5:169735023-169735423	chr7:83687943-83688343
chr1:247039033-247039433	chr12:46950365-46950765	chr17:74442201-74442601	chr21:15940541-15940941	chr5:17000944-17001344	chr7:92347373-92347773
chr1:24968989-24969389	chr12:47011067-47011467	chr17:76338185-76338585	chr21:17909456-17909856	chr5:17024818-17025218	chr7:92746907-92747307
chr1:25559171-25559571	chr12:47358263-47358663	chr17:76836375-76836775	chr21:20830338-20830738	chr5:172124263-172124663	chr7:93800510-93800910
chr1:26947061-26947461	chr12:49658963-49659363	chr17:77826681-77827081	chr21:20847568-20847968	chr5:172201868-172202268	chr7:98000644-98001044
chr1:27191895-27192295	chr12:50636083-50636483	chr17:78236791-78237191	chr21:30581348-30581748	chr5:172234510-172234910	chr7:98700650-98701050
chr1:27976829-27977229	chr12:51157384-51157784	chr17:78518573-78518973	chr21:34379747-34380147	chr5:172437633-172438033	chr7:99063360-99063760
chr1:27993956-27994356	chr12:52540867-52541267	chr17:78785670-78786070	chr21:34697255-34697655	chr5:172881646-172882046	chr7:99942203-99942603
chr1:28201541-28201941	chr12:52821182-52821582	chr17:79819286-79819686	chr21:34752817-34753217	chr5:173229547-173229947	chr8:100905470-100905870
chr1:28295536-28295936	chr12:53365596-53365996	chr17:80292778-80293178	chr21:35028449-35028849	chr5:173281566-173281966	chr8:101539566-101539966
chr1:28701108-28701508	chr12:53871722-53872122	chr17:80296781-80297181	chr21:36202991-36203391	chr5:174256842-174257242	chr8:102054773-102055173
chr1:28928541-28928941	chr12:56754282-56754682	chr17:9120756-9121156	chr21:36214182-36214582	chr5:175839470-175839870	chr8:102817306-102817706
chr1:32110167-32110567	chr12:56920654-56921054	chr18:10525365-10525765	chr21:38738995-38739395	chr5:176921421-176921821	chr8:104426962-104427362
chr1:32701311-32701711	chr12:57913942-57914342	chr18:11119293-11119693	chr21:38740691-38741091	chr5:177923903-177924303	chr8:106200045-106200445
chr1:33254606-33255006	chr12:59494982-59495382	chr18:12033250-12033650	chr21:40159818-40160218	chr5:179159265-179159665	chr8:108316201-108316601
chr1:33399505-33399905	chr12:60089143-60089543	chr18:12287824-12288224	chr21:40177129-40177529	chr5:179264013-179264413	chr8:116588770-116589170
chr1:33474283-33474683	chr12:64263604-64264004	chr18:19019672-19020072	chr21:42783051-42783451	chr5:179899551-179899951	chr8:119068532-119068932
chr1:33490021-33490421	chr12:64853423-64853823	chr18:20290790-20291190	chr21:44596876-44597276	chr5:18394011-18394411	chr8:120218771-120219171
chr1:35247885-35248285	chr12:66582562-66582962	chr18:20985974-20986374	chr21:44727549-44727949	chr5:18745938-18746338	chr8:122012213-122012613
chr1:36500441-36500841	chr12:69201947-69202347	chr18:2605888-2606288	chr21:44768930-44769330	chr5:1963066-1963466	chr8:124049555-124049955
chr1:36851808-36852208	chr12:69221454-69221854	chr18:2641786-2642186	chr21:45177967-45178367	chr5:2094361-2094761	chr8:124287285-124287685
chr1:37920529-37920929	chr12:69860979-69861379	chr18:2642496-2642896	chr22:18189074-18189474	chr5:21152319-21152719	chr8:124399653-124400053
chr1:37933813-37934213	chr12:70698720-70699120	chr18:27081540-27081940	chr22:18893297-18893697	chr5:21680148-21680548	chr8:125488679-125489079
chr1:38015795-38016195	chr12:71055021-71055421	chr18:27082309-27082709	chr22:19095495-19095895	chr5:23013508-23013908	chr8:125878262-125878662
chr1:40626932-40627332	chr12:71209406-71209806	chr18:29034242-29034642	chr22:21922006-21922406	chr5:23466933-23467333	chr8:128352655-128353055
chr1:41884770-41885170	chr12:747092-747492	chr18:2960547-2960947	chr22:26150597-26150997	chr5:26872066-26872466	chr8:128867559-128867959

chr1:42092223-42092623	chr12:755274-755674	chr18:2980782-2981182	chr22:28262462-28262862	chr5:28726544-28726944	chr8:130697695-130698095
chr1:44387278-44387678	chr12:76376876-76377276	chr18:3594866-3595266	chr22:29169074-29169474	chr5:31939633-31940033	chr8:130994334-130994734
chr1:44812891-44813291	chr12:81171706-81172106	chr18:3624507-3624907	chr22:29195758-29196158	chr5:32020361-32020761	chr8:131054747-131055147
chr1:44951092-44951492	chr12:8210949-8211349	chr18:41268824-41269224	chr22:29811088-29811488	chr5:35881033-35881433	chr8:131074443-131074843
chr1:45019076-45019476	chr12:82751739-82752139	chr18:43628312-43628712	chr22:30987821-30988221	chr5:35918899-35919299	chr8:132916666-132917066
chr1:47082504-47082904	chr12:8394883-8395283	chr18:43631723-43632123	chr22:31569333-31569733	chr5:35922117-35922517	chr8:136469235-136469635
chr1:47223104-47223504	chr12:84352986-84353386	chr18:43830046-43830446	chr22:35697097-35697497	chr5:36665243-36665643	chr8:13670655-13671055
chr1:47271852-47272252	chr12:8834184-8834584	chr18:43830744-43831144	chr22:36634938-36635338	chr5:37198060-37198460	chr8:141601615-141602015
chr1:47277788-47278188	chr12:88692803-88693203	chr18:47794452-47794852	chr22:38902014-38902414	chr5:37273212-37273612	chr8:141841551-141841951
chr1:48223831-48224231	chr12:89319292-89319692	chr18:48556459-48556859	chr22:39378485-39378885	chr5:37668917-37669317	chr8:142011841-142012241
chr1:51535857-51536257	chr12:96794850-96795250	chr18:52490099-52490499	chr22:39916211-39916611	chr5:38871958-38872358	chr8:142037073-142037473
chr1:52030031-52030431	chr12:97300557-97300957	chr18:52495580-52495980	chr22:40672313-40672713	chr5:39014263-39014663	chr8:142141124-142141524
chr1:53600876-53601276	chr13:100157857-100158257	chr18:52613118-52613518	chr22:41032386-41032786	chr5:39015050-39015450	chr8:142146399-142146799
chr1:54232539-54232939	chr13:101045267-101045667	chr18:55672014-55672414	chr22:41238867-41239267	chr5:39074155-39074555	chr8:143896859-143897259
chr1:55181560-55181960	chr13:109923436-109923836	chr18:56349932-56350332	chr22:41652001-41652401	chr5:39661288-39661688	chr8:144100159-144100559
chr1:56046139-56046539	chr13:110036457-110036857	chr18:56549226-56549626	chr22:41776988-41777388	chr5:40641657-40642057	chr8:144640070-144640470
chr1:5777363-5777763	chr13:110597479-110597879	chr18:56558872-56559272	chr22:42084901-42085301	chr5:40679086-40679486	chr8:17104466-17104866
chr1:59084202-59084602	chr13:113862976-113863376	chr18:57552078-57552478	chr22:42239443-42239843	chr5:40798010-40798410	chr8:17362726-17363126
chr1:59250302-59250702	chr13:114065395-114065795	chr18:57566996-57567396	chr22:42253130-42253530	chr5:43313829-43314229	chr8:19471564-19471964
chr1:61946637-61947037	chr13:114829914-114830314	chr18:60189686-60190086	chr22:43093295-43093695	chr5:43422530-43422930	chr8:22102915-22103315
chr1:62190465-62190865	chr13:20438367-20438767	chr18:65085226-65085626	chr22:43463223-43463623	chr5:43514821-43515221	chr8:22246464-22246864
chr1:62901572-62901972	chr13:21654087-21654487	chr18:68001328-68001728	chr22:44020875-44021275	chr5:43935267-43935667	chr8:22249507-22249907
chr1:64979636-64980036	chr13:25086354-25086754	chr18:72166707-72167107	chr22:45977061-45977461	chr5:44808979-44809379	chr8:22926090-22926490
chr1:65047803-65048203	chr13:25485487-25485887	chr18:74812946-74813346	chr22:46465032-46465432	chr5:485736-486136	chr8:23418965-23419365
chr1:65468591-65468991	chr13:25497143-25497543	chr18:77725292-77725692	chr22:50219879-50220279	chr5:52657702-52658102	chr8:28177245-28177645
chr1:65502690-65503090	chr13:25892235-25892635	chr18:8800885-8801285	chr22:50629008-50629408	chr5:5422428-5422828	chr8:28227046-28227446
chr1:6661434-6661834	chr13:32889152-32889552	chr19:10381278-10381678	chr22:50720635-50721035	chr5:54929859-54930259	chr8:29971073-29971473
chr1:6761199-6761599	chr13:42039687-42040087	chr19:1128436-1128836	chr22:50765774-50766174	chr5:5523497-5523897	chr8:38255368-38255768
chr1:68092376-68092776	chr13:43500805-43501205	chr19:1151745-1152145	chr22:50969409-50969809	chr5:56538962-56539362	chr8:39770345-39770745
chr1:68148958-68149358	chr13:44238815-44239215	chr19:1177462-1177862	chr22:50975194-50975594	chr5:58592659-58593059	chr8:49503028-49503428
chr1:68151110-68151510	chr13:44847046-44847446	chr19:1180152-1180552	chr22:50983172-50983572	chr5:58886854-58887254	chr8:49540145-49540545
chr1:68315864-68316264	chr13:45914975-45915375	chr19:11877899-11878299	chr3:100264604-100265004	chr5:58957699-58958099	chr8:50730334-50730734
chr1:70671337-70671737	chr13:49142140-49142540	chr19:12405162-12405562	chr3:100279874-100280274	chr5:64062255-64062655	chr8:52874963-52875363
chr1:72587875-72588275	chr13:49600726-49601126	chr19:14229155-14229555	chr3:101395435-101395835	chr5:66187643-66188043	chr8:59508784-59509184
chr1:76320971-76321371	chr13:49618524-49618924	chr19:1438317-1438717	chr3:101546702-101547102	chr5:66190095-66190495	chr8:64081102-64081502
chr1:78805957-78806357	chr13:49619816-49620216	chr19:14530233-14530633	chr3:10215144-10215544	chr5:66306874-66307274	chr8:71286995-71287395
chr1:8064233-8064633	chr13:50069713-50070113	chr19:14999428-14999828	chr3:10234109-10234509	chr5:66611247-66611647	chr8:74252908-74253308
chr1:80840505-80840905	chr13:50265844-50266244	chr19:15054176-15054576	chr3:103998758-103999158	chr5:6722261-6722661	chr8:75085858-75086258
chr1:86849026-86849426	chr13:51850403-51850803	chr19:15054847-15055247	chr3:105070310-105070710	chr5:6732800-6733200	chr8:81397241-81397641

chr1:89239474-89239874	chr13:52988657-52989057	chr19:16189192-16189592	chr3:111663475-111663875	chr5:68485676-68486076	chr8:81398387-81398787
chr1:89450944-89451344	chr13:53239817-53240217	chr19:16254707-16255107	chr3:11267275-11267675	chr5:708417-708817	chr8:90741755-90742155
chr1:89488143-89488543	chr13:75727640-75728040	chr19:16959607-16960007	chr3:119691816-119692216	chr5:72117712-72118112	chr8:91107313-91107713
chr1:89530825-89531225	chr13:76056823-76057223	chr19:17416601-17417001	chr3:122084607-122085007	chr5:72251271-72251671	chr8:9756890-9757290
chr1:90320728-90321128	chr13:76063841-76064241	chr19:17887222-17887622	chr3:122660007-122660407	chr5:72418298-72418698	chr8:98192967-98193367
chr1:92197743-92198143	chr13:76111931-76112331	chr19:18475340-18475740	chr3:123451661-123452061	chr5:72794172-72794572	chr9:101010637-101011037
chr1:9256554-9256954	chr13:76123567-76123967	chr19:2165401-2165801	chr3:124704370-124704770	chr5:73537080-73537480	chr9:107510172-107510572
chr1:9295356-9295756	chr13:76279146-76279546	chr19:23577868-23578268	chr3:125351662-125352062	chr5:73611955-73612355	chr9:110302018-110302418
chr1:934862-935262	chr13:76346413-76346813	chr19:24270027-24270427	chr3:125635722-125636122	chr5:74147758-74148158	chr9:111034499-111034899
chr1:935790-936190	chr13:77566576-77566976	chr19:2817133-2817533	chr3:125653560-125653960	chr5:74409215-74409615	chr9:111872500-111872900
chr1:940487-940887	chr13:77879748-77880148	chr19:2818716-2819116	chr3:126227572-126227972	chr5:74967771-74968171	chr9:112811372-112811772
chr1:94158749-94159149	chr13:79905954-79906354	chr19:2900307-2900707	chr3:127310059-127310459	chr5:77656105-77656505	chr9:112877933-112878333
chr1:94167755-94168155	chr13:81690761-81691161	chr19:33072370-33072770	chr3:127771540-127771940	chr5:77996055-77996455	chr9:114244976-114245376
chr1:94220317-94220717	chr13:83262547-83262947	chr19:35158303-35158703	chr3:128597844-128598244	chr5:81074718-81075118	chr9:116648803-116649203
chr1:95194863-95195263	chr13:88322932-88323332	chr19:3558060-3558460	chr3:133682035-133682435	chr5:8457558-8457958	chr9:117050740-117051140
chr1:95633528-95633928	chr13:90167225-90167625	chr19:36036112-36036512	chr3:134189073-134189473	chr5:8907031-8907431	chr9:123453688-123454088
chr1:95634684-95635084	chr13:91097734-91098134	chr19:3655522-3655922	chr3:135232997-135233397	chr5:89407288-89407688	chr9:123688453-123688853
chr1:99304343-99304743	chr13:92876927-92877327	chr19:38794932-38795332	chr3:135789608-135790008	chr5:90326816-90327216	chr9:127155455-127155855
chr10:100163817-100164217	chr13:93242723-93243123	chr19:3969134-3969534	chr3:13642662-13643062	chr5:90676337-90676737	chr9:13014254-13014654
chr10:101743826-101744226	chr13:96256561-96256961	chr19:39888693-39889093	chr3:136649650-136650050	chr5:91841570-91841970	chr9:130213177-130213577
chr10:1034500-1034900	chr13:97302583-97302983	chr19:40790094-40790494	chr3:142166478-142166878	chr5:92900141-92900541	chr9:130276125-130276525
chr10:103949162-103949562	chr13:99161199-99161599	chr19:40939366-40939766	chr3:146236034-146236434	chr5:93768660-93769060	chr9:130818320-130818720
chr10:104153770-104154170	chr14:100842032-100842432	chr19:43239994-43240394	chr3:146268568-146268968	chr5:93805507-93805907	chr9:130860790-130861190
chr10:104155656-104156056	chr14:102438462-102438862	chr19:43337007-43337407	chr3:148897315-148897715	chr5:94966672-94967072	chr9:131133644-131134044
chr10:104364331-104364731	chr14:103672742-103673142	chr19:43355306-43355706	chr3:148963681-148964081	chr5:95158877-95159277	chr9:131321491-131321891
chr10:104562595-104562995	chr14:103723088-103723488	chr19:43379262-43379662	chr3:150089319-150089719	chr5:95237556-95237956	chr9:132245444-132245844
chr10:104677654-104678054	chr14:103870310-103870710	chr19:43403202-43403602	chr3:152878714-152879114	chr5:96270271-96270671	chr9:132254455-132254855
chr10:104892977-104893377	chr14:23340259-23340659	chr19:43686097-43686497	chr3:156201618-156202018	chr5:97751212-97751612	chr9:132504856-132505256
chr10:104985417-104985817	chr14:24681972-24682372	chr19:43705360-43705760	chr3:156252739-156253139	chr6:104981816-104982216	chr9:132522178-132522578
chr10:104990498-104990898	chr14:27897244-27897644	chr19:44178752-44179152	chr3:156878107-156878507	chr6:109195720-109196120	chr9:133326908-133327308
chr10:105654793-105655193	chr14:30206782-30207182	chr19:44205044-44205444	chr3:168759614-168760014	chr6:111549496-111549896	chr9:133617763-133618163
chr10:108412380-108412780	chr14:30663516-30663916	chr19:45149034-45149434	chr3:169172850-169173250	chr6:111978739-111979139	chr9:134714716-134715116
chr10:111662942-111663342	chr14:35183122-35183522	chr19:45682242-45682642	chr3:169684874-169685274	chr6:112144680-112145080	chr9:137258331-137258731
chr10:112116795-112117195	chr14:35875727-35876127	chr19:45879783-45880183	chr3:170626816-170627216	chr6:11341692-11342092	chr9:139428003-139428403
chr10:112187796-112188196	chr14:39644015-39644415	chr19:46117682-46118082	chr3:171897983-171898383	chr6:1139772-1140172	chr9:139826848-139827248
chr10:112612271-112612671	chr14:39901328-39901728	chr19:46684358-46684758	chr3:172304050-172304450	chr6:114040438-114040838	chr9:139980669-139981069
chr10:114135675-114136075	chr14:44785733-44786133	chr19:47103872-47104272	chr3:172346956-172347356	chr6:1141956-1142356	chr9:139981652-139982052
chr10:115439780-115440180	chr14:50335185-50335585	chr19:48017949-48018349	chr3:176566377-176566777	chr6:117095397-117095797	chr9:140136272-140136672
chr10:11594195-11594595	chr14:50429695-50430095	chr19:48233903-48234303	chr3:178788316-178788716	chr6:117784713-117785113	chr9:140206159-140206559

chr10:116279845-116280245	chr14:52578421-52578821	chr19:4831745-4832145	chr3:179169408-179169808	chr6:121655658-121656058	chr9:140506600-140507000
chr10:11928553-11928953	chr14:55032160-55032560	chr19:49375920-49376320	chr3:180124081-180124481	chr6:12483576-12483976	chr9:14317770-14318170
chr10:12028249-12028649	chr14:55239612-55240012	chr19:50144354-50144754	chr3:18176240-18176640	chr6:12491954-12492354	chr9:14320640-14321040
chr10:120839948-120840348	chr14:55505794-55506194	chr19:53141254-53141654	chr3:182971180-182971580	chr6:125211804-125212204	chr9:1474596-1474996
chr10:121006290-121006690	chr14:56662611-56663011	chr19:54483789-54484189	chr3:183004322-183004722	chr6:125628746-125629146	chr9:16119968-16120368
chr10:124053161-124053561	chr14:57735751-57736151	chr19:54511411-54511811	chr3:183088257-183088657	chr6:12593178-12593578	chr9:16959400-16959800
chr10:124180126-124180526	chr14:68034098-68034498	chr19:5690228-5690628	chr3:183875161-183875561	chr6:126138080-126138480	chr9:21827994-21828394
chr10:131343359-131343759	chr14:70233997-70234397	chr19:59066441-59066841	chr3:184053834-184054234	chr6:131286386-131286786	chr9:22055136-22055536
chr10:134227415-134227815	chr14:71339107-71339507	chr19:8008846-8009246	chr3:185035010-185035410	chr6:131456893-131457293	chr9:2838156-2838556
chr10:14091308-14091708	chr14:73268276-73268676	chr2:10183701-10184101	chr3:187596792-187597192	chr6:137539963-137540363	chr9:31304225-31304625
chr10:14127318-14127718	chr14:76051974-76052374	chr2:101923886-101924286	chr3:187912351-187912751	chr6:138050551-138050951	chr9:31304742-31305142
chr10:14995775-14996175	chr14:78266650-78267050	chr2:102589967-102590367	chr3:188394618-188395018	chr6:138132606-138133006	chr9:32432794-32433194
chr10:15243233-15243633	chr14:83630782-83631182	chr2:102758714-102759114	chr3:189948275-189948675	chr6:138144843-138145243	chr9:33263598-33263998
chr10:22845949-22846349	chr14:83892087-83892487	chr2:10716701-10717101	chr3:190023748-190024148	chr6:138187664-138188064	chr9:33290528-33290928
chr10:24754781-24755181	chr14:91580769-91581169	chr2:10798344-10798744	chr3:192675424-192675824	chr6:138198628-138199028	chr9:33329596-33329996
chr10:25110720-25111120	chr14:93570794-93571194	chr2:110349077-110349477	chr3:194392876-194393276	chr6:139938860-139939260	chr9:34233133-34233533
chr10:27443742-27444142	chr14:95945825-95946225	chr2:111435658-111436058	chr3:197439321-197439721	chr6:140519439-140519839	chr9:34254290-34254690
chr10:29452531-29452931	chr15:101611075-101611475	chr2:113299432-113299832	chr3:197445443-197445843	chr6:14271653-14272053	chr9:35072078-35072478
chr10:29495199-29495599	chr15:101835495-101835895	chr2:118804611-118805011	chr3:197456844-197457244	chr6:143935692-143936092	chr9:36731847-36732247
chr10:32097435-32097835	chr15:30136132-30136532	chr2:121377448-121377848	chr3:197518046-197518446	chr6:14665490-14665890	chr9:37290996-37291396
chr10:3214515-3214915	chr15:30282892-30283292	chr2:122479778-122480178	chr3:23366627-23367027	chr6:146865345-146865745	chr9:38171044-38171444
chr10:33552477-33552877	chr15:31780539-31780939	chr2:122494962-122495362	chr3:23709567-23709967	chr6:146867964-146868364	chr9:75733626-75734026
chr10:34024035-34024435	chr15:33141972-33142372	chr2:128284120-128284520	chr3:23780833-23781233	chr6:149555286-149555686	chr9:77635439-77635839
chr10:3828058-3828458	chr15:36720767-36721167	chr2:128568391-128568791	chr3:23790475-23790875	chr6:15063114-15063514	chr9:88356359-88356759
chr10:3848781-3849181	chr15:40415426-40415826	chr2:135676332-135676732	chr3:23955337-23955737	chr6:158034083-158034483	chr9:89686029-89686429
chr10:3867820-3868220	chr15:41913860-41914260	chr2:137919701-137920101	chr3:23987820-23988220	chr6:158383360-158383760	chr9:90407936-90408336
chr10:3894064-3894464	chr15:42263621-42264021	chr2:141261241-141261641	chr3:24527416-24527816	chr6:159065206-159065606	chr9:92002877-92003277
chr10:3910680-3911080	chr15:42349346-42349746	chr2:141867769-141868169	chr3:28337434-28337834	chr6:160114862-160115262	chr9:93754621-93755021
chr10:45655267-45655667	chr15:42783394-42783794	chr2:147602200-147602600	chr3:28389484-28389884	chr6:160390865-160391265	chr9:93763001-93763401
chr10:45656110-45656510	chr15:44083829-44084229	chr2:147961530-147961930	chr3:29395207-29395607	chr6:160394819-160395219	chr9:94587047-94587447
chr10:46205869-46206269	chr15:45011449-45011849	chr2:151474775-151475175	chr3:30341231-30341631	chr6:16237078-16237478	chr9:94600659-94601059
chr10:62297045-62297445	chr15:45356389-45356789	chr2:151477208-151477608	chr3:31959238-31959638	chr6:169852077-169852477	chr9:94696567-94696967
chr10:63541450-63541850	chr15:45748583-45748983	chr2:151482225-151482625	chr3:33341250-33341650	chr6:18259101-18259501	chr9:94713883-94714283
chr10:71919426-71919826	chr15:49087022-49087422	chr2:152146167-152146567	chr3:33705047-33705447	chr6:20168400-20168800	chr9:94784767-94785167
chr10:71921514-71921914	chr15:51401178-51401578	chr2:152708583-152708983	chr3:33840040-33840440	chr6:20445122-20445522	chr9:95857395-95857795
chr10:71992665-71993065	chr15:51406739-51407139	chr2:153553238-153553638	chr3:36999719-37000119	chr6:20543535-20543935	chr9:95896420-95896820
chr10:73734756-73735156	chr15:52024220-52024620	chr2:153963450-153963850	chr3:38193061-38193461	chr6:22892416-22892816	chr9:96213406-96213806
chr10:79504278-79504678	chr15:52121354-52121754	chr2:157287394-157287794	chr3:38195168-38195568	chr6:257280-257680	chr9:97411599-97411999
chr10:79909322-79909722	chr15:52154785-52155185	chr2:161138512-161138912	chr3:40158124-40158524	chr6:26286038-26286438	chr9:98972229-98972629

chr10:90843870-90844270	chr15:55095920-55096320	chr2:164067355-164067755	chr3:40494332-40494732	chr6:26440627-26441027	chrX:108780099-108780499
chr10:91061571-91061971	chr15:55681260-55681660	chr2:165300616-165301016	chr3:40498687-40499087	chr6:27446884-27447284	chrX:118018173-118018573
chr10:91087314-91087714	chr15:58920714-58921114	chr2:172873220-172873620	chr3:41200811-41201211	chr6:2791054-2791454	chrX:118018807-118019207
chr10:91132592-91132992	chr15:59312757-59313157	chr2:172959846-172960246	chr3:43811893-43812293	chr6:2854173-2854573	chrX:123198200-123198600
chr10:91133870-91134270	chr15:59826561-59826961	chr2:175862724-175863124	chr3:45593157-45593557	chr6:34203985-34204385	chrX:128766051-128766451
chr10:95187454-95187854	chr15:59827343-59827743	chr2:178076806-178077206	chr3:46152963-46153363	chr6:35606561-35606961	chrX:128796776-128797176
chr10:99092985-99093385	chr15:59981167-59981567	chr2:187350409-187350809	chr3:46755041-46755441	chr6:35886831-35887231	chrX:130951136-130951536
chr10:99186227-99186627	chr15:60875344-60875744	chr2:190739280-190739680	chr3:46976233-46976633	chr6:36721076-36721476	chrX:15353039-15353439
chr10:99447481-99447881	chr15:60952885-60953285	chr2:190798248-190798648	chr3:47204767-47205167	chr6:37059051-37059451	chrX:21799436-21799836
chr11:102317755-102318155	chr15:61091215-61091615	chr2:191708980-191709380	chr3:48060113-48060513	chr6:37207914-37208314	chrX:23760884-23761284
chr11:10324924-10325324	chr15:63188437-63188837	chr2:191746974-191747374	chr3:48343057-48343457	chr6:3768075-3768475	chrX:23791306-23791706
chr11:110243787-110244187	chr15:63526182-63526582	chr2:191748846-191749246	chr3:48732509-48732909	chr6:41755414-41755814	chrX:23837803-23838203
chr11:110586747-110587147	chr15:63664122-63664522	chr2:191878621-191879021	chr3:49383908-49384308	chr6:41888862-41889262	chrX:32471713-32472113
chr11:111101349-111101749	chr15:63768741-63769141	chr2:192067296-192067696	chr3:49449021-49449421	chr6:42067709-42068109	chrX:37706385-37706785
chr11:119992398-119992798	chr15:66084838-66085238	chr2:192068988-192069388	chr3:49851127-49851527	chr6:423131-423531	chrX:44937002-44937402
chr11:122056907-122057307	chr15:67409486-67409886	chr2:193132010-193132410	chr3:5166192-5166592	chr6:43595376-43595776	chrX:45207547-45207947
chr11:125461978-125462378	chr15:68406546-68406946	chr2:194052720-194053120	chr3:57790006-57790406	chr6:43597280-43597680	chrX:67724277-67724677
chr11:12728024-12728424	chr15:70087244-70087644	chr2:197343478-197343878	chr3:57969693-57970093	chr6:43885231-43885631	chrX:67791793-67792193
chr11:13095322-13095722	chr15:74818135-74818535	chr2:19735916-19736316	chr3:57985708-57986108	chr6:44307281-44307681	chrX:70823159-70823559
chr11:13237251-13237651	chr15:74822931-74823331	chr2:201247497-201247897	chr3:64075700-64076100	chr6:45572582-45572982	chrX:83756841-83757241
chr11:13359403-13359803	chr15:75092169-75092569	chr2:201832549-201832949	chr3:64123961-64124361	chr6:45582337-45582737	chrX:9450136-9450536
chr11:14541564-14541964	chr15:75400590-75400990	chr2:201993272-201993672	chr3:72242721-72243121	chr6:47260143-47260543	chrX:9834369-9834769
chr11:16760049-16760449	chr15:75404887-75405287	chr2:202015598-202015998	chr3:73115632-73116032	chr6:47382497-47382897	

Note: The notation "chromosome:start_position-end_position" signifies the peak location.

Table S3. All primer and siRNA sequences used in this study

Names	Species	Forward primer	Reverse primer
ISG15	human	5'-CGCAGATCACCCAGAAGATCG-3'	5'-TTCGTCGCATTTGTCCACCA-3'
IFI27	human	5'-TGCTCTCACCTCATCAGCAGT-3'	5'-CACAACTCCTCCAATCACAACT-3'
IRF7	human	5'-CCCAGCAGGTAGCATTCCC-3'	5'-GCAGCAGTTCCTCCGTGTAG-3'
MX1	human	5'- AGCGGGATCGTGACCAGAT-3'	5'-TGACCTTGCCTCTCCACTTATC-3'
OAS1	human	5'-TGTCCAAGGTGGTAAAGGGTG-3'	5'-CCGGCGATTTAACTGATCCTG-3'
OAS2	human	5'-CTCAGAAGCTGGGTTGGTTTAT-3'	5'-ACCATCTCGTCGATCAGTGTC-3'
OAS3	human	5'-GAAGGAGTTCGTAGAGAAGGCG-3'	5'-CCCTTGACAGTTTTCAGCACC-3'
OASL	human	5'-CTGATGCAGGAACTGTATAGCAC-3'	5'-CACAGCGTCTAGCACCTCTT-3'
cGAS	human	5'-CACGAAGCCAAGACCTCCG-3'	5'-GTCGCACTTCAGTCTGAGCA-3'
STING	human	5'-CCAGAGCACACTCTCCGGTA-3'	5'-CGCATTTGGGAGGGAGTAGTA-3'
RIGI	human	5'-CTGGACCCTACCTACATCCTG-3'	5'-GGCATCCAAAAAGCCACGG-3'
MDA5	human	5'-TCGAATGGGTATTCCACAGACG-3'	5'-GTGGCGACTGTCCTCTGAA-3'
IRF3	human	5'-AGAGGCTCGTGATGGTCAAG-3'	5'-AGGTCCACAGTATTCTCCAGG-3'
IFIT1	human	5'-GCGCTGGGTATGCGATCTC-3'	5'-CAGCCTGCCTTAGGGGAAG-3'
IFIT2	human	5'-AAGCACCTCAAAGGGCAAAAC-3'	5'-TCGGCCCATGTGATAGTAGAC-3'
IFIT3	human	5'-TCAGAAGTCTAGTCACTTGGGG-3'	5'-ACACCTTCGCCCTTTCATTTC-3'
EGFP	human	5'-GTGACCACCCTGACCTACG-3'	5'-TCAGCTCGATGCGGTTCAC-3'
GAPDH	human	5'-GGAGCGAGATCCCTCCAAAAT-3'	5'-GGCTGTTGTCATACTTCTCATGG-3'

Names	Species	siRNA sequence
IRF3-1	human	GAUCUGAUUACCUUCACGGAA
IRF3-2	human	CCCUUCAUUGUAGAUCUGAUU
IRF7-1	human	GCUGGACGUGACCAUCAUGUA
IRF7-2	human	CCCGAGCUGCACGUUCCUAUA
RIGI-1	human	GGAUUGUUACAGUUCAGAAUU
RIGI-2	human	GCCCUGUUUUUAUACACUUUU
MDA5-1	human	GUAACAUUGUUAUCCGUUAUU
MDA5-2	human	GGUGUAAGAGAGCUACUAAUU
STING-1	human	GGAUUCGAACUUACAAUCAUU
STING-2	human	GGUCAUAUUACAUCGGAUAUU
cGAS-1	human	GGAAGAAAUUAACGACAUUUU
cGAS-2	human	CCAACACUCGUGCAUAUUAUU

Table S4. FPKM values of the cGAS and IFI16 genes in the wild-type HEK293T and HCT116 cell lines

Gene	HEK293T_WT_rep1	HEK293T_WT_rep2	HEK293T_WT_rep3	HCT116_WT_rep1	HCT116_WT_rep2	HCT116_WT_rep3
cGAS	0.019739833	0	0	0	0	0
IFI16	0.030985646	0.015843108	0.039085144	0.016557617	0	0.015505598

Note: "WT" denotes wild type.
